# Structural basis of stepwise proton sensing-mediated GPCR activation

**DOI:** 10.1038/s41422-025-01092-w

**Published:** 2025-04-11

**Authors:** Xiaolei Yue, Li Peng, Shenhui Liu, Bingjie Zhang, Xiaodan Zhang, Hao Chang, Yuan Pei, Xiaoting Li, Junlin Liu, Wenqing Shui, Lijie Wu, Huji Xu, Zhi-Jie Liu, Tian Hua

**Affiliations:** 1 iHuman Institute, ShanghaiTech University, Shanghai, China; 2 School of Life Science and Technology, ShanghaiTech University, Shanghai, China; 3 Department of Rheumatology and Immunology, Changzheng Hospital, Second Military Medical University, Shanghai, China; 4 School of Clinical Medicine, Tsinghua University, Beijing, China; 5 Peking-Tsinghua Center for Life Sciences, Tsinghua University, Beijing, China

**Keywords:** Electron microscopy, Cell signalling

## Abstract

The regulation of pH homeostasis is crucial in many biological processes vital for survival, growth, and function of life. The pH-sensing G protein-coupled receptors (GPCRs), including GPR4, GPR65 and GPR68, play a pivotal role in detecting changes in extracellular proton concentrations, impacting both physiological and pathological states. However, comprehensive understanding of the proton sensing mechanism is still elusive. Here, we determined the cryo-electron microscopy structures of GPR4 and GPR65 in various activation states across different pH levels, coupled with G_s_, G_q_ or G_13_ proteins, as well as a small molecule NE52-QQ57-bound inactive GPR4 structure. These structures reveal the dynamic nature of the extracellular loop 2 and its signature conformations in different receptor states, and disclose the proton sensing mechanism mediated by networks of extracellular histidine and carboxylic acid residues. Notably, we unexpectedly captured partially active intermediate states of both GPR4–G_s_ and GPR4–G_q_ complexes, and identified a unique allosteric binding site for NE52-QQ57 in GPR4. By integrating prior investigations with our structural analysis and mutagenesis data, we propose a detailed atomic model for stepwise proton sensation and GPCR activation. These insights may pave the way for the development of selective ligands and targeted therapeutic interventions for pH sensing-relevant diseases.

## Introduction

The pH homeostasis is crucial for human health, and effective proton sensing is essential for monitoring and maintaining the optimal conditions necessary for functionality and survival.^1,2^ Among the over 800 known human G protein-coupled receptors (GPCRs), three specific receptors — GPR4, GPR65 (T-cell death-associated gene 8 or TDAG8), and GPR68 (ovarian cancer G protein-coupled receptor 1 or OGR1) — were identified as proton (H^+^ ion) sensors regulating signaling within acidic microenvironments.^3^ Discovered in 2003, these receptors are predominantly expressed in lymphoid tissues, macrophages, and innate immune cells,^4,5^ making them as the promising therapeutic targets due to their involvement in acid–base balance and inflammation-related pathologies.^6,7^ The three proton-sensing GPCRs exhibit different responses to extracellular pH variations, and each receptor has distinct preferences for G protein-mediated signal transduction.^8^ GPR4, in particular, is responsive to protons and engages with various G proteins, playing critical roles in angiogenesis,^9,10^ endothelial cell inflammation,^11^ and cancer cell metastasis suppression.^12^ Conversely, GPR65 is activated at relatively lower pH levels, primarily signals via the G_s_ pathway, and is implicated in chronic inflammatory diseases such as inflammatory bowel disease and certain tumors.^6,13–16^

Despite being the pH sensors, the exploration for small-molecule ligands targeting GPR4 and GPR65 is pursued. The reported antagonist NE52-QQ57, specific to GPR4, has shown promise in preclinical models.^17–19^ Moreover, previous studies implicated that some lipids, lysophosphatidylcholine (LPC) and sphingosylphosphorylcholine, could be putative modulators for GPR4.^20–23^ However, only a few ligands have been identified so far,^24^ and the modulation of these receptors by small molecules or lipids needs further exploration.

Though previous studies, including evolutionary genetics, mutagenesis and prediction modeling^25^ proposed various mechanisms of proton sensing in GPCRs, the absence of experimental three-dimensional (3D) structures limited the comprehensive understanding of these receptors. In this study, we present the cryo-electron microscopy (cryo-EM) structures of GPR4 and GPR65 in their active states at varied pH levels, coupled with different G proteins, along with a small molecule NE52-QQ57-induced inactive GPR4 structure, which provides important references for in-depth analysis of the activation process of proton-sensing GPCRs.

## Results

### Structures of GPR4–G_s_ complexes activated at different pH levels

**Fig. 1:**
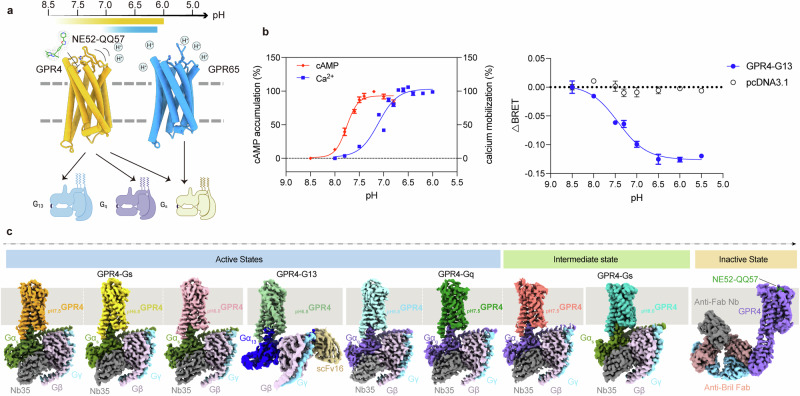
Overall structures of GPR4–G protein complexes and NE52-QQ57-bound inactive GPR4. **a** Schematic diagram showing GPR4- and GPR65-mediated activation of G protein signaling pathways across different extracellular pH ranges. **b** Concentration-response curves of pH-induced GPR4 activation measured by the cellular cAMP accumulation for G_s_ pathway, FLIPR calcium Ca^2+^ assay for the G_q_ pathway (left), and bioluminescence resonance energy transfer (BRET) assay for G_13_ pathway (right). Data are means ± SEM from three independent experiments (*n* = 3). **c** Cryo-EM density maps of _pH7.5_GPR4–G_s_, _pH6.8_GPR4–G_s_, _pH6.0_GPR4–G_s_, _pH6.8_GPR4–G_13_, _pH7.5_GPR4–G_q_-state-1, _pH7.5_GPR4–G_q_-state-2, _pH8.0_GPR4–G_s_ and NE52-QQ57–GPR4-Bril-Fab complexes.

To examine the proton activation profiles of GPR4, we performed the cAMP accumulation assay to measure the receptor’s activity across different pH levels. The pH-dependent G_s_-cAMP assay was performed within a pH range of 8.5 to 6.5, and the results revealed that the proton concentration-response curve for GPR4 reaches a maximum intracellular cAMP accumulation plateau at pH range of 7.5 to 6.8 (Fig. 1a, b). These findings also guided the sample preparation conditions for subsequent structural elucidations. To enhance GPR4 protein expression yields, the endoglucanase H protein (PDB: 2CIT) was fused to the N-terminus of the wild-type (WT) GPR4. This modification maintained the proton sensitivity comparable to that of the WT receptor (Supplementary information, Fig. S1a). The GPR4–G_s_ complex was then assembled through co-expression in insect cells, followed by purification at pH values of 7.5, 6.8 and 6.0, respectively (Supplementary information, Fig. S2). Using cryo-EM single particle analysis, the structures of _pH7.5_GPR4–G_s_, _pH6.8_GPR4–G_s_ and _pH6.0_GPR4–G_s_ complexes were determined at global resolutions of 2.9 Å, 3.1 Å and 3.1 Å, respectively (Fig. 1c; Supplementary information, Fig. S2 and Table S1). In addition, we constituted the GPR4–G_s_ complex at a lower proton concentration (pH 8.0) and successfully solved the structure of _pH8.0_GPR4–G_s_ (Fig. 1c; Supplementary information, Fig. S5 and Table S1).

Structurally, the seven-transmembrane (7TM) bundles in GPR4–G_s_ complexes at pH values ranging from 6.0 to 7.5 exhibit similar folds (Supplementary information, Fig. S1b, c). The extracellular loop 2 (ECL2) adopts a conformation analogous to that of the resolved δ-branch GPCRs, which forms a β-sheet hairpin structure (Supplementary information, Fig. S1d). Unexpectedly, in the _pH8.0_GPR4–G_s_ structure, the ECL2 adopts a distinct conformation (Supplementary information, Fig. S1b, c), which will be discussed later. Of note, two disulfide bonds (C90^3.25^–C168^ECL2^ and C9^N-term^–C258^7.25^) are observed to stabilize the receptor’s extracellular domain (Supplementary information, Fig. S1c), with the disulfide bond C9^N-term^–C258^7.25^ unique for GPR4. Mutagenesis data indicated that the disruption of each disulfide bond impaired the receptor’s proton responding capability, as evidenced by a rightward shift in the EC_50_ curve and an altered maximum activation response (*E*_max_) compared to the WT receptor in our cAMP accumulation assay, whereas the disulfide bond linking TM3 and ECL2 (C90^3.25^–C168^ECL2^) showed a more significant effect (Supplementary information, Fig. S1e).

### Structures of G_q_- or G_13_-coupled GPR4 complexes

To elucidate the G protein coupling versatility of GPR4, we determined the structures of chimeric miniG_s/q_-coupled GPR4 activated at pH 7.5 (_pH7.5_GPR4–G_q_) and pH 6.8 (_pH6.8_GPR4–G_q_), and miniG_13/iN_-coupled GPR4 activated at pH 6.8 (pH_6.8_GPR4–G_13_). These structures were solved at global resolutions of 2.9 Å, 2.7 Å and 3.4 Å, respectively (Fig. 1c; Supplementary information, Fig. S3 and Table S1). For clarity, Gα_q_ and Gα_13_ denote their respective chimeric constructs throughout this report.

**Fig. 2:**
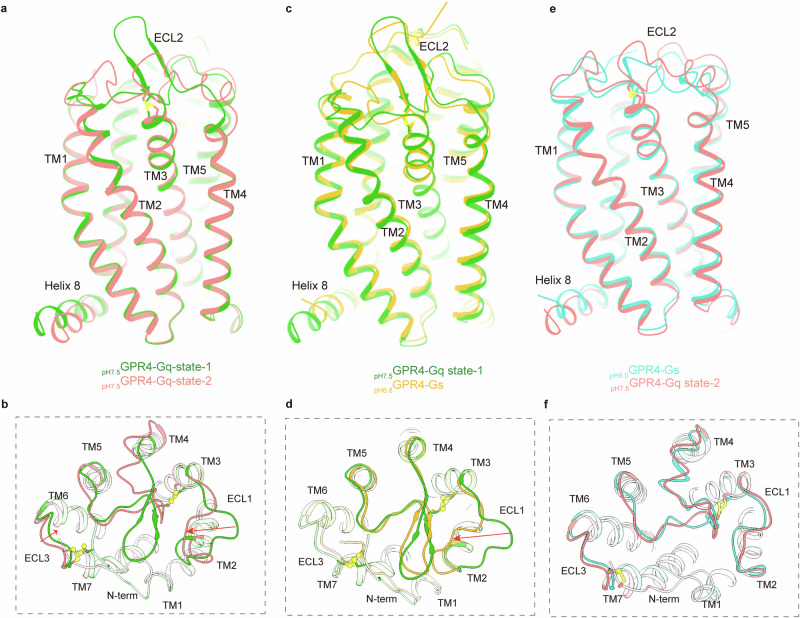
Structural comparison of _pH7.5_GPR4–G_q_ in two states, _pH7.5_GPR4–G_s_ and _pH8.0_GPR4–G_s_ complex. **a**, **b** Structural superposition of _pH7.5_GPR4–G_q_ in state-1 and state-2 in side view (**a**) and in the extracellular region including disulfide bonds (**b**). **c**, **d** Structural superposition of _pH7.5_GPR4–G_q_-state-1 and _pH7.5_GPR4–G_s_ in side view (**c**) and in the extracellular region including disulfide bonds (**d**). **e**, **f** Structural superposition of _pH7.5_GPR4–G_q_-state-2 and _pH8.0_GPR4–G_s_ in side view (**e**) and in the extracellular region including disulfide bonds (**f**).

Notably, two distinct groups of cryo-EM particles were obtained from one batch of _pH7.5_GPR4–G_q_ samples, and the resolved structures were named _pH7.5_GPR4–G_q_-state-1 and _pH7.5_GPR4–G_q_-state-2, respectively (Fig. 1c; Supplementary information, Fig. S3g–m). Comparative analysis on the two structures reveals significant differences in the conformation of extracellular domain, especially ECL2 (Fig. 2a). In _pH7.5_GPR4–G_q_-state-1 structure, ECL2 adopts a typical β-sheet hairpin configuration observed in G_s_- (pH 6.0, 6.8 and 7.5) or G_13_-coupled active GPR4 (Fig. 2c); however, in _pH7.5_GPR4–G_q_-state-2, ECL2 resembles that in the _pH8.0_GPR4–G_s_ structure (Fig. 2e, f), which is packed more horizontally into the 7TM bundle core accompanied by the varied conformations of ECL1, ECL3 and the extracellular ends of TMs 2, 4–7 (Fig. 2a, b; Supplementary information, Video S1). Both structures maintain the integrity of the two pairs of disulfide bonds (Fig. 2b). However, the _pH7.5_GPR4–G_q_-state-1 structure is more similar to the fully active _pH6.8_GPR4–G_q_ or _pH6.8_GPR4–G_s_ structure, including the extracellular conformation, indicating that it is in an active state (Fig. 2c, d). In contrast, _pH7.5_GPR4–G_q_-state-2 and _pH8.0_GPR4–G_s_ are probably in intermediate states captured at the initial stage of receptor activation at pH 7.5 and pH 8.0, respectively.

Furthermore, additional long tube-like densities were observed in the cavity formed by TMs 3–5 in the G_q_-coupled GPR4 structures (Supplementary information, Fig. S4a), suggesting potential lipid binding. To identify potential lipid candidates, we performed mass spectrometry (MS) analysis on the _pH7.5_GPR4–G_q_ samples (see “Materials and Methods”). The results indicated that the LPC 16:1 is abundant and the preferred lipid, featuring a phosphocholine head group and a 16-carbon fatty acid chain (Supplementary information, Fig. S4d, e). However, further experiments are needed to confidently identify the lipid-like molecule.

### Allosteric binding mode of NE52-QQ57 in GPR4

**Fig. 3:**
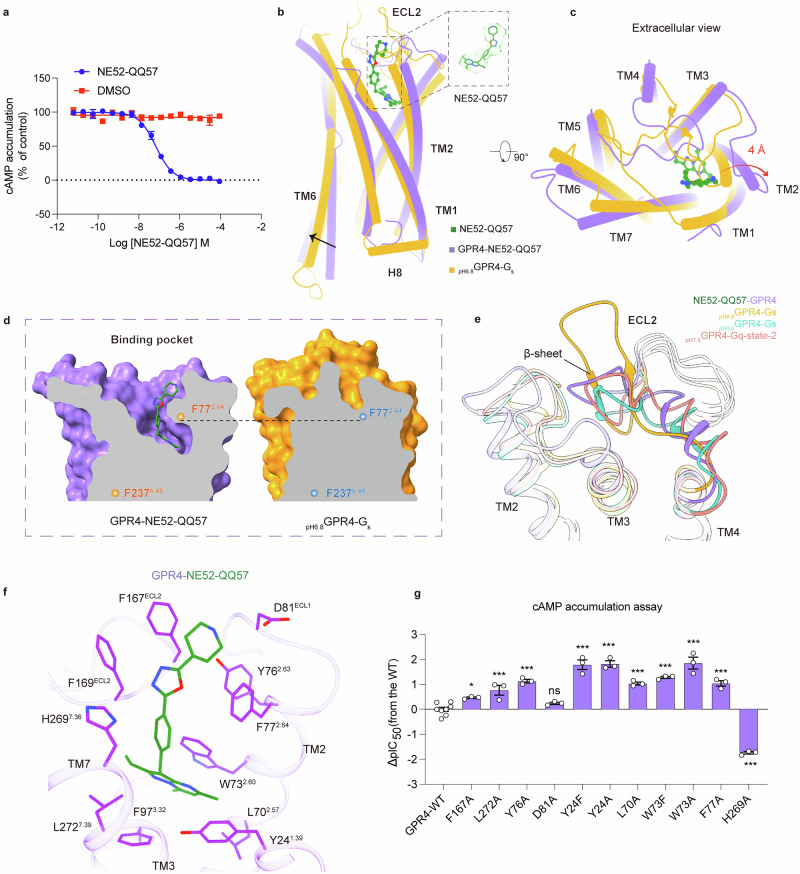
Overall architecture of the inactive NE52-QQ57–GPR4 complex structure. **a** NE52-QQ57 induced a dose-dependent antagonist response at pH 7.5, as determined by the cAMP accumulation assay. Data are from three independent experiments (*n* = 3). **b**, **c** Structural comparison of _pH6.8_GPR4–G_s_ and NE52-QQ57-bound GPR4 in side view (**b**) and extracellular view (**c**). NE52-QQ57 in the binding pocket is highlighted and the electron density for NE52-QQ57 is shown in the dotted box (**b**). **d** Surface cutaway side views comparing NE52-QQ57-bound inactive GPR4 and active GPR4 structures. The conserved toggle-switch F237^6.48^ and F77^2.64^, part of the NE52-QQ57-binding pocket, are used as the references. **e** The conformation changes of ECL2 in NE52-QQ57–GPR4, _pH6.8_GPR4–G_s_, _pH8.0_GPR4–G_s_ and _pH7.5_GPR4–G_q_-state-2. **f** Interactions between NE52-QQ57 and key residues from GPR4. **g** Mutagenesis effects of the key residues interacting with NE52-QQ57, measured by cAMP accumulation assay. Bars represent differences in calculated potency (pIC_50_) for each mutant shown as percentage of the maximum in WT. Statistical differences between WT and mutants were determined by two-sided, one-way ANOVA with Tukey’s test. *P < 0.05; **P < 0.01; ***P < 0.001. Data are presented as the means ± SEM of three independent experiments performed in triplicate.

NE52-QQ57 was reported to selectively inhibit the activity of GPR4,^17^ showing an IC_50_ of 72 nM in our cAMP accumulation assay (Fig. 3a). Following extensive optimization, we determined the cryo-EM structure of the NE52-QQ57-bound GPR4 using the previously described antibody-BRIL strategy^26^ (Materials and Methods; Fig. 1c; Supplementary information, Fig. S5 and Table S1). In comparison with the GPR4–G_s_ and GPR4–G_q_ complexes, the NE52-QQ57-bound receptor adopts an inactive conformation, characterized by the converged cytoplasmic segment of TM6 and the inactive states of the activation-related motifs, typical of class A GPCRs (Fig. 3b). Notably, the extracellular end of TM2 bends outward creating a binding pocket for NE52-QQ57 near the extracellular surface formed by TMs 1–3 and TM7 (Fig. 3c). The canonical orthosteric pocket in active GPR4 structure is predominantly occupied by several bulky residues (Fig. 3d), and consequently, NE52-QQ57 binds near the TM2 side in the 7TM core (Fig. 3c). Remarkably, the conformation of ECL2 in the NE52-QQ57-bound structure undergoes substantial alterations, resembling those observed in the _pH8.0_GPR4–G_s_ and _pH7.5_GPR4–G_q_-state-2 structures (Fig. 3e). Further characterization of structural features indicates that the conformation of ECL2 with a horizontal short loop hairpin in NE52-QQ57-bound GPR4, _pH8.0_GPR4–G_s_ and _pH7.5_GPR4–G_q_-state-2 structures represents an inactive state.

In the NE52-QQ57-bound GPR4 structure, NE52-QQ57 adopts an L-shaped conformation within the binding pocket, and its bicyclic core forms π–π interactions with W73^2.60^ and F97^3.32^, as well as a polar interaction with Y24^1.39^ and a hydrophobic interaction with L272^7.39^ (Fig. 3f). Mutation of W73^2.60^ or Y24^1.39^ to alanine or phenylalanine reduces the inhibition activity of NE52-QQ57 (Fig. 3g). Moreover, the piperidine moiety of NE52-QQ57 is oriented towards the extracellular surface, forming hydrophobic interactions with F77^2.64^, F167^ECL2^, F169^ECL2^ and H269^7.36^ (Fig. 3f). The F77^2.64^A mutation also decreases the inhibition activity of NE52-QQ57 on GPR4 (Fig. 3g). The discovery of this unique binding pocket in GPR4 may facilitate the design of more potent and selective small-molecule ligands targeting proton-sensing GPCRs.

Of note, the lipid-like density located in the cavity formed by TMs 3–5 is not observed in the NE52-QQ57-bound inactive structure due to the rotation of TMs 3–5 and conformational change of ICL2 (Supplementary information, Fig. S4b). In the inactive structure, E145^4.53^ rotates into the 7TM core, disrupting its interaction with the lipid (Supplementary information, Fig. S4c). MS analysis of the NE52-QQ57-bound GPR4 samples showed significantly lower levels of LPC compared to those in _pH7.5_GPR4–G_q_ samples (Supplementary information, Fig. S4e). These findings indicate a potential role of the conserved residue E^4.53^ in lipid modulation of proton-sensing GPCRs.

### Mechanism of proton sensing in GPR4

**Fig. 4:**
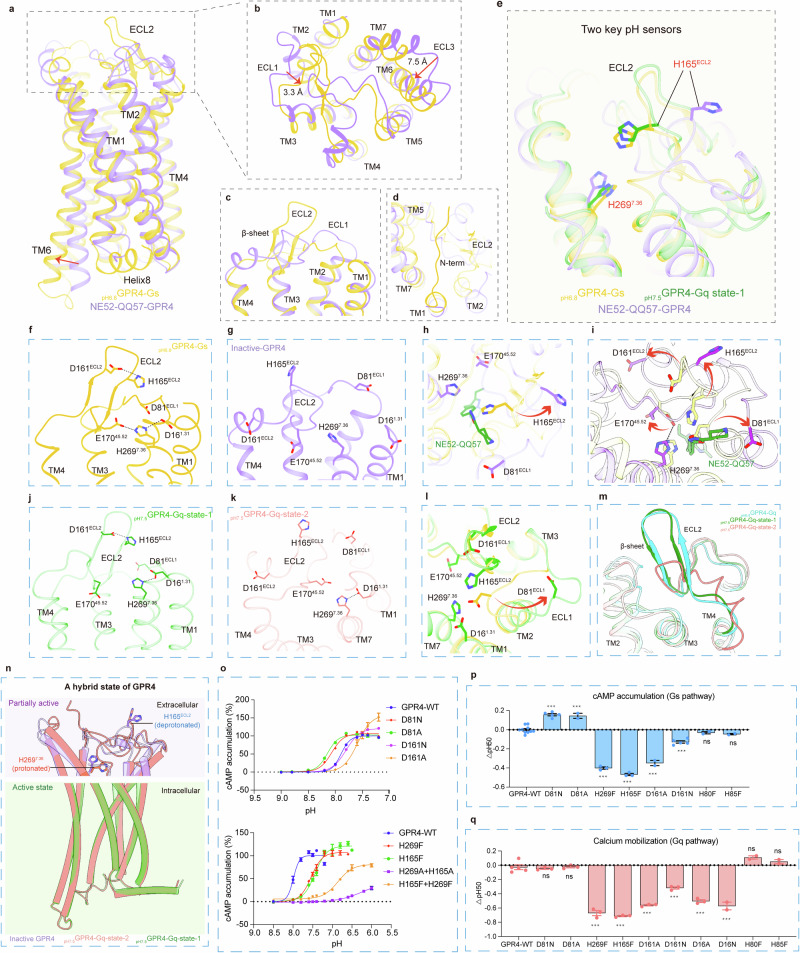
Proton sensing mechanism of GPR4. **a**–**d** The conformational changes in the key proton-sensing networks between active and inactive GPR4 structures. Structural superposition of _pH6.8_GPR4–G_s_ and NE52-QQ57–GPR4 in side view (**a**) and in the extracellular region (**b**). The conformational changes in ECLs (**c**) and N-terminus (**d**) between active and inactive GPR4 structures. **e** Two key proton-sensing histidine residues H165^ECL2^ and H269^7.36^ in GPR4. **f**, **g** Detailed interaction network around H165^ECL2^ and H269^7.36^ in _pH6.8_GPR4–G_s_ (**f**) and inactive GPR4 (**g**) structures (hydrogen bonds are marked by dashed lines). **h** H165^ECL2^ rotates outwards in the inactive GPR4 structure. **i** Antagonist binding disrupts interaction networks of key proton-sensing residues. **j**, **k** Detailed interaction network around H165^ECL2^ and H269^7.36^ in _pH7.5_GPR4–G_q_-state-1 (**j**), _pH7.5_GPR4–G_q_-state-2 (**k**) structures (hydrogen bonds are marked by dashed lines). **l** The conformational changes of the key proton-sensing networks between _pH6.8_GPR4–G_s_ and _pH7.5_GPR4–G_q_-state-1 structures. **m** ECL2 conformations in _pH8.0_GPR4–G_s_, _pH7.5_GPR4–G_q_-state-1 and _pH7.5_GPR4–G_q_-state-2 structures. **n** Schematic presentation of hybrid combination of the active intracellular domain and the partially active extracellular domain of GPR4 in _pH7.5_GPR4–G_q_-state-2 structure. **o**–**q** Effects of mutations in key proton-sensing residues in GPR4 measured by GloSensor assay (**o**, **p**) and FLIPR calcium Ca^2+^ assay (**q**). pH_50_ represents the calculated half-maximal effective response to protons for each mutant. Data are means ± SEM from three independent experiments (*n* = 3). Statistical differences between WT and mutants were determined by two-sided, one-way ANOVA with Tukey’s test. *P < 0.05; **P < 0.01; ***P < 0.001.

Structural comparison analysis on G_s_- or G_q_-coupled GPR4 at different pH levels and the NE52-QQ57-bound inactive GPR4 reveals significant conformational changes of GPR4 at different states. In detail, from inactive to active states, ECL1 and ECL3 converge towards the extracellular core by approximately 3.3 Å and 7.5 Å (using W83^ECL1^ and W256^ECL3^ as references), respectively, while the intracellular end of TM6 swings outward by 8.9 Å (referenced by Q217^6.28^), a hallmark of class A GPCR activation (Fig. 4a, b). More dramatic conformation changes occur in ECL2 and the N-terminus. ECL2 transforms from a short helix linked to a horizontal short loop hairpin to a vertically protruding long β-sheet hairpin (Figs. 3e and 4c). Simultaneously, the N-terminus transitions from a disordered state to an ordered long loop overlaying the extracellular cleft of the active GPR4 (Fig. 4d; Supplementary information, Video S2).

Previous research underscored the role of histidine residues in proton-sensing GPCRs, and GPR4 harbors eight histidine residues in the extracellular domain.^27^ Examination of the inactive and active GPR4 structures, along with the protonation calculations, revealed clusters of histidine and carboxylic acid residues that constitute a complex extracellular proton-sensing network (Fig. 4e). To further elucidate the proton sensing mechanism in GPR4, the _pH6.8_GPR4–G_s_ complex structure was selected for more in-depth analysis due to its better EM density map and higher structure quality (Supplementary information, Fig. S7a, b). p*K*_a_ calculations of the histidine residues in _pH6.8_GPR4–G_s_ revealed that H165^ECL2^ and H269^7.36^ are the key pH sensors, with a protonation ratio of ~99.9% (HIP state, both nitrogen in the imidazole ring were protonated) (Supplementary information, Fig. S7d). In the _pH6.8_GPR4–G_s_ structure, protonated H165^ECL2^ forms close interactions with residues D81^ECL1^ and D161^ECL2^ (Fig. 4f), and the H165^ECL2^F or D161^ECL2^N/A mutations reduced pH sensitivity, as evidenced by a lower half-maximal effective pH (pH_50_) (Fig. 4o, p). In addition, the protonated H269^7.36^ forms a hydrogen bond with E170^45.52^, and polar interactions with D16^1.31^ and D81^ECL1^, linking two histidine clusters within a proton-sensing network (Fig. 4f). Consistently, mutation of H269^7.36^F resulted in significant pH_50_ reduction (Fig. 4o, p). In addition, the molecular dynamics (MD) simulation results indicate the stable interactions between the key interaction pairs (Supplementary information, Fig. S7c). Moreover, in the NE52-QQ57-bound inactive GPR4, NE52-QQ57 disrupts the native proton-sensing network by driving the outward rotation of H165^ECL2^, and pushing H269^7.36^ out of its original location (Fig. 4g–i; Supplementary information, Video S3).

### The stepwise proton sensing in GPR4

In the GPR4–G_q_ complexes, the receptor at pH 6.8 (_pH6.8_GPR4–G_q_) adopts a similar conformation to that in the _pH6.8_GPR4–G_s_ complex. However, in the _pH7.5_GPR4–G_q_-state-1 structure, ECL1 shows an outward shift, possibly due to the different modulation at the initial activation stage between G_s_ and G_q_ coupling (Fig. 4l). Most of the proton-sensing framework in the _pH6.8_GPR4–G_s_ structure is retained in the _pH7.5_GPR4–G_q_-state-1 complex, including the critical proton-sensing interactions involving H269^7.36^ and D16^1.31^(Fig. 4f, j). While H165^ECL2^ still interacts with D161^ECL2^, the outward shift of ECL1 causes D81^ECL1^ to fall outside the interacting range of H165^ECL2^ (Fig. 4j, l, q).

The _pH7.5_GPR4–G_q_-state-2 complex exhibits a hybrid structure that combines a partially functional extracellular proton-sensing domain with an active transmembrane and intracellular receptor body (Fig. 4n). In detail, the inactive conformation of ECL2 hampers the proton-sensing capability of the H165^ECL2^ cluster, yet H269^7.36^ remains responsive to proton (Fig. 4k, n; Supplementary information, Video S4). This responsiveness supports GPR4 activation, as indicated by the active conformation of the activation-related motifs and the coupling of G_q_. Therefore, it is plausible to propose a stepwise proton sensing mechanism for GPR4–G_q_. Specifically, at pH 7.5, H269^7.36^ is protonated, facilitating close interactions with surrounding carboxylic acidic residues, which subsequently triggers a downstream signaling cascade that shifts the receptor into an active state (Fig. 4j, k). In this phase, the co-existing active and partially active GPR4–G_q_ complex particles yield _pH7.5_GPR4–G_q_-state-1 and _pH7.5_GPR4–G_q_-state-2 structures separately. In the subsequent phase, with the increasing proton concentration, ECL2 transforms into an active conformation, fully restoring the proton-sensing network and resulting in a fully active state (Fig. 4m; Supplementary information, Video S4).

The structural comparison analysis revealed similarities between the _pH8.0_GPR4–G_s_ and _pH7.5_GPR4–G_q_-state-2 structures, including the inactive conformation of ECL2, which impairs the proton sensing capability of H165^ECL2^ (Figs. 2e, f, 4m).

These findings highlight that proton sensing and signaling in GPR4 are orchestrated by a network of histidine and carboxylic acid residues in a stepwise manner across both G_s_ and G_q_ signaling pathways. This is supported by the observation that no single mutation of the proton-sensing histidine residues completely abolished GPR4 activation. However, a double mutation of H269^7.36^F/A and H165^ECL2^F/A almost disrupted the activation (Fig. 4o).

### Activation of GPR4

**Fig. 5:**
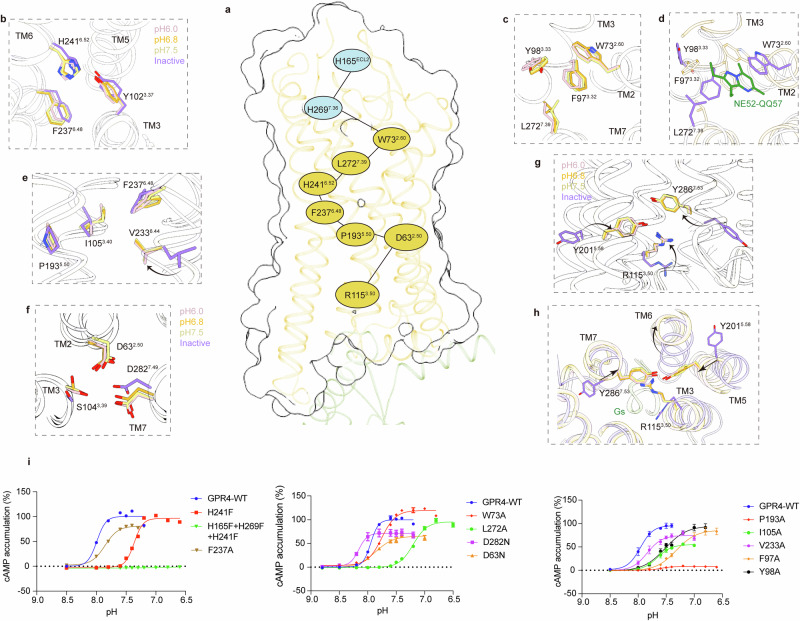
Activation mechanism of GPR4. **a** The key proton-sensing residues (light blue) and signaling activation-associated residues (yellow) constituted networks in superimposed structures of GPR4–G_s_ at pH 7.5, pH 6.8 and pH 6.0, as well as the inactive GPR4. **b** Rearrangements of the interactions around the conserved residue H241^6.52^. **c**, **d** The hydrophobic core in superimposed structures of GPR4–G_s_ at pH 7.5, pH 6.8 and pH 6.0 (**c**), which is disrupted by antagonist binding (**d**). **e**–**h** Rearrangements of the PIF motif and toggle-switch residues (**e**), the sodium-binding pocket (**f**) and the NPxxY motif (**g**, **h**). **i** Concentration-response curves showing effects of the key residue mutations on GPR4 activation measured by cellular cAMP accumulation for G_s_ pathway. Data are presented as the means ± SEM of three independent experiments performed in triplicate.

In GPR4 structure, H241^6.52^, a conserved residue in proton-sensing GPCRs, is situated one helix turn above the toggle-switch residue F237^6.48^ (Fig. 5a, b), and functions as a crucial proton-singling network node in GPR4 (Supplementary information, Fig. S6e). H241^6.52^F mutation impacts the receptor’s pH responsiveness (Fig. 5i), demonstrating its essential role in signal transduction probably in pH-sensing GPCRs. Additionally, a triple mutant, combining H165^ECL2^A/F, H269^7.36^A/F and H241^6.52^A/F, completely abolished proton-induced activation of GPR4 (Fig. 5i). Concurrently, residues W73^2.60^, F97^3.32^, Y98^3.33^ and L272^7.39^ establish a hydrophobic core that stabilizes the active conformation of TM2, TM3 and TM7 (Fig. 5c), which is disrupted in the NE52-QQ57-bound structure (Fig. 5d).

Activation in class A GPCRs typically begins at the transmission switch (P^5.50^I^3.40^F^6.44^ motif and F^6.48^) and the sodium (Na^+^)-binding pocket (2.50, 3.39, 7.45 and 7.49)^28^ (Fig. 5e, f). Unlike typical class A GPCRs that feature tryptophan at position 6.48, δ-branch GPCRs, including GPR4, exhibit F/Y/L substitutions, with F237^6.48^ exhibiting a downward shift in active structures (Fig. 5b). The F237^6.48^A mutation impaired GPR4 activation (Fig. 5i). Additionally, the proposed TRIAD core^25^ residues (D63^2.50^ and D282^7.49^) conserved across proton-sensing GPCRs, contribute to the Na^+^-binding site. The activation-associated structural rearrangement leads to the collapse of the Na^+^-binding pocket and initiates the movement of TM7 towards active conformations (Fig. 5f). In our functional assay, D63^2.50^N mutation decreased proton’s efficacy on GPR4, indicating the important role of D63^2.50^ in activation (Fig. 5i). Moreover, the mutation of D282^7.49^N in the N(D)PxxY motif increased the pH_50_ but decreased the efficacy of the receptor, supporting the involvement of D282^7.49^ in activation processes (Fig. 5i). Concurrently, Y201^5.58^ and Y286^7.53^ rotate into the transmembrane bundle, forming polar interactions with R115^3.50^ when the receptor transforms from an inactive to an active state (Fig. 5g). This shift enhances the packing between TM3 and TM5 as well as that between TM3 and TM7, while loosening TM3–TM6 interactions, ultimately promoting TM6’s outward movement and enabling G protein coupling at the cytosolic site in active GPR4 structures (Fig. 5h).

### Structure of the GPR65–G_s_ complex

**Fig. 6:**
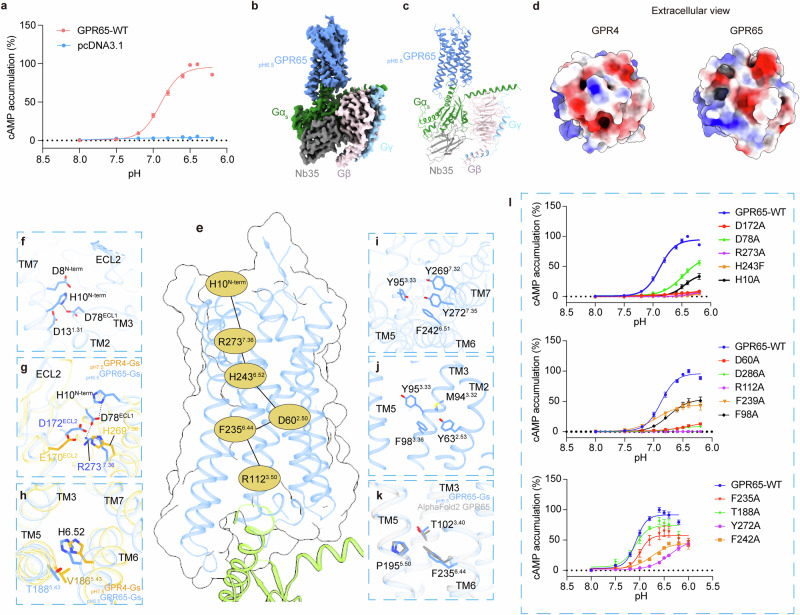
Proton sensing and activation in GPR65. **a** Concentration-response curves of pH-induced GPR65 activation measured by cellular cAMP accumulation for G_s_ pathway. Data are means ± SEM from three independent experiments (*n* = 3). **b**, **c** Cryo-EM density map (**b**) and ribbon representation (**c**) of the _pH6.5_GPR65–G_s_ complex. **d** Electrostatic potential mapped onto the solvent-accessible surface of GPR4 and GPR65 highlighting highly negative cavities in the extracellular domain. **e** The key proton-sensing residues (light blue) and signaling activation-associated residues (yellow) constituted networks in superimposed structures of _pH6.5_GPR65–G_s_ and the inactive-like GPR65 predicted by AlphaFold2. **f** Proton-sensing network around H10^N-term^. **g** Proton-sensing network around R273^7.36^ and its comparison with that of H269^7.36^ in _pH7.5_GPR4–G_s_. **h** Structural comparison of the interaction involving H6.52 between GPR65 and GPR4. **i**, **j** The polar and hydrophobic interaction cores in GPR65. **k** Rearrangement of the PIF motif. **l** Concentration-response curves for key residue mutants of GPR65 across the extracellular pH spectrum measured by GloSensor assay. Data are means ± SEM from three independent experiments (*n* = 3).

GPR65 exhibits maximal activity at pH 6.5 in our cAMP accumulation assay (Fig. 6a). To stabilize the GPR65–G_s_ complex, engineered GPR65 (Materials and Methods) was co-expressed with miniG_s_^29^ in insect cells. The structure of the GPR65–G_s_ complex, activated at pH 6.5 (_pH6.5_GPR65–G_s_), was solved at an overall nominal resolution of 3.3 Å (Fig. 6b, c; Supplementary information, Fig. S5k–o, f and Table S1). Notably, the overall density map quality is adequate for fitting the receptor and the G protein (Supplementary information, Fig. S5o), except for the extracellular region, in which the density is relatively weaker than that of GPR4. Thus, the modeling of ECL2 was guided by AlphaFold2-predicted GPR65 structure.^30^ In the predicted model, GPR65’s ECL2 also folds into a β-sheet hairpin conformation and potentially forms disulfide bonds with TM3 and the N-terminus (C87^3.25^–C170^ECL2^ and C5^N-term^–C160^ECL2^, respectively), differing from those in GPR4 (Supplementary information, Fig. S6a–c). Our mutagenesis studies confirmed the important roles of these disulfide bonds in maintaining receptor activity, with mutation of C160^ECL2^A or C87^3.25^A almost abolishing GPR65’s proton-sensing capability (Supplementary information, Fig. S1f).

Compared with GPR4 structures, the 7TM bundle and the receptor–G_s_ protein interface are highly conserved (Supplementary information, Fig. S6a). However, GPR65 exhibits a more expansive and conformationally flexible extracellular cavity than GPR4, suggesting unique receptor dynamics (Fig. 6d).

### Proton sensing mechanism shared by GPR65 and GPR4

GPR65 has fewer exposed extracellular histidine residues (H10^N-term^, H14^1.32^, H254^6.63^ and H261^7.24^) than GPR4 (Supplementary information, Fig. S6d). Notably, in the _pH6.5_GPR65–G_s_ structure, the H10^N-term^ is proximal to D8^N-term^, D13^1.31^ and D78^ECL1^, facilitating the formation of an interaction network (Fig. 6f). Mutation of H10^N-term^A or D78^ECL1^A impaired GPR65’s proton-sensing efficacy, shifting its optimal pH to a lower range (Fig. 6l). Additionally, R273^7.36^ of GPR65, corresponding to GPR4’s H269^7.36^, appears to coordinate with residues D78^ECL1^ and D172^ECL2^ (E170^ECL2^ in GPR4), forming another proton-sensing cluster (Fig. 6g). Alanine mutations of D172^ECL2^ or R273^7.36^ almost abolished the proton-sensing activity of GPR65 (Fig. 6l).

Comparatively, GPR4 and GPR65 share similarities but also diverge in their proton sensing mechanisms, with GPR65’s reduced sensitivity potentially linked to fewer histidine–carboxylic acid residue interactions. However, the conserved carboxylic acid residue D/E, from TM1 and ECL2, encompassing the H^7.36^/R^7.36^ residue, underscores their significance in proton detection in both receptors (Supplementary information, Fig. S6e).

Regarding the conserved H^6.52^ residue in both receptors, H243^6.52^ in GPR65 forms a polar interaction with T188^5.43^ at pH 6.5 (Fig. 6h). Consistently, H243^6.52^F mutation significantly diminishes GPR65’s activation by protons (Fig. 6l). The protonation-induced activation of GPR65 is further stabilized by hydrophobic and π–π interaction cores (Fig. 6e, i, j), with one formed by Y269^7.32^, Y272^7.35^, Y95^3.33^ and F242^6.51^ (Fig. 6i), and the other consisting of Y63^2.53^, M94^3.32^, Y95^3.33^ and F98^3.36^ (Fig. 6j). Moreover, the activation-related motifs P^5.50^I^3.40^F^6.44^, N(D)P^7.50^xxY and DR^3.50^Y in GPR65, adopt similar conformations to those observed in GPR4 (Fig. 6k).

### G protein coupling modes in GPR4 and GPR65

**Fig. 7:**
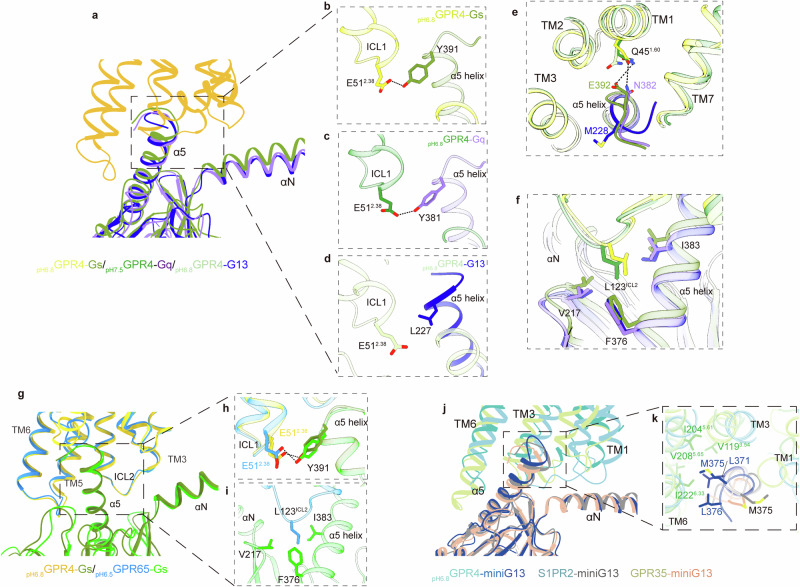
Coupling modes of GPR4 with G_s_, G_q_ and G_13_ and that of GPR65 with G_s_. **a** Structural alignment of the α5 helix (Gα_s_, Gα_q_ and Gα_13_) binding modes in GPR4. **b**–**d** The hydrogen-bonding interactions between α5 helix in Gα_s_ (**b**) or Gα_q_ (**c**) with E51^2.38^ in GPR4, which is absent in Gα_13_ (**d**). **e** The hydrogenbonding interactions between α5 helix in Gα_s_ or Gα_q_ with Q45^1.60^ in GPR4, but absent in Gα_13_. **f** The conserved hydrophobic interaction core between ICL2 and Gα subunits. **g**–**i** Structural comparison of the α5 helix (Gα_s_) binding modes in GPR4 and GPR65 (**g**), the hydrogen-bonding interaction between Y391 from Gα_s_ and E51^2.38^ (**h**) and the hydrophobic interaction core between ICL2 and Gα_s_ (**i**). **j**, **k** Structural comparison of the overall receptor–G protein interaction mode (**j**) and the methionine pocket (**k**) in the GPR4–G_13_ complex with other reported G_13_-coupled class A GPCRs, including S1PR2 (PDB: 7T6B) and GPR35 (PDB: 8H8J).

GPR4 demonstrates versatile coupling capabilities with various Gα protein subfamilies, including G_s_, G_13_ and G_q/11_.^31–33^ The resolved structures complexing with Gα_s_, Gα_13_, or Gα_q_ elucidate diverse G protein coupling modes (Fig. 7a). These structures reveal the binding modes across different Gα subunits, characterized by the hydrophobic and several polar or hydrogen bonding interactions previously observed involving the α5 and αN helices (Fig. 7b–f; Supplementary information, Fig. S6f, g). Specifically, in the GPR4–G_s_ complex structures, E392 and Y391 in the α5 helix of Gα_s_ form polar contacts with Q45^1.60^ and E51^2.38^ from TMs 1–2 and ICL1 region of GPR4 (Fig. 7b, e). While similar interactions are observed in the GPR4–Gα_q_ complex (Fig. 7c, e), they are absent in the GPR4–Gα_13_ structure (Fig. 7d). The intracellular conformational variations result in distinct receptor–G protein interactions on their coupling interface, with GPR4–Gα_s_ forming the largest interface area of 1230 Å^2^, followed by GPR4–Gα_13_ (1017 Å^2^), and GPR4–Gα_q_ (1114 Å^2^). Notably, L123^34.51^ in ICL2 is buried in a hydrophobic groove formed by the αN–β1 junction, the β2–β3 loop, and the α5 helix of the G protein, in G_s_-, G_q_- and G_13_-coupled structures (Fig. 7f). Furthermore, the GPR65–G_s_ binding interface closely resembles that of GPR4–G_s_ (Fig. 7g–i).

Comparative structural analysis of G_13_-coupled class A GPCRs, including GPR35 (PDB: 8H8J)^34^ and sphingosine-1-phosphate receptor 2 (S1PR2, PDB: 7T6B),^35^ indicates a similar Gα_13_ binding mode. In the GPR4–G_13_ complex, the α5 and αN helices align more closely with those in S1PR2–G_13_ structure (Fig. 7j). Notably, M375 in Gα_12/13_, a residue that varies among G protein subtypes, points towards a unique “methionine pocket” proposed in GPR35 structure^34^ (Fig. 7k). The detailed structural insights offer a foundation for designing ligands that preferentially activate specific signaling pathways in GPR4.

## Discussion

This study elucidates the pH sensing mechanisms within GPCRs, revealing crucial biological processes that facilitate cellular communication and enable organisms to adapt to their environments. Taking GPR4 and its evolutionary ancestor GPR65 as models, we uncovered the distinct yet shared proton-sensing networks formed by key histidine and carboxylic acid residues. These networks coordinate proton signaling and receptor activation, orchestrating responses to environmental changes. Remarkably, our study captures two distinct states in both G_s_- and G_q_-coupled GPR4 complexes, representing active and partially active intermediate states.

Further comparative structural analysis, using the NE52-QQ57-bound inactive GPR4 structure as a reference, delineates the sequential processes of proton sensing initiation, signal propagation, and receptor activation. At the proper pH level, protonation of H269^7.36^ in GPR4 establishes hydrogen bonds and polar interactions with neighboring carboxylic acidic residues, triggering downstream signaling that activates the receptor and facilitates G protein coupling. As pH shifts towards more acidic conditions, H269^7.36^ protonation-induced conformational changes drive the reorganization of the extracellular loops and N-terminus, with ECL2 playing a pivotal role in transforming the extracellular domain into an active state. This culminates in the full activation of GPR4, while the entire proton-sensing network, including the functional H165^ECL2^–carboxylic acidic residue cluster is restored. This observation and analysis led us to propose a stepwise model for proton sensing and activation in GPR4.

Moreover, the structures of GPR4 coupled with G_s_, G_q_, or G_13_, as well as GPR65 coupled with G_s_, provide a detailed map of the receptor–G protein interface, showcasing how subtle shifts in intracellular loops and transmembrane helix orientations contribute to the specificity and efficiency of downstream signaling pathways.

Collectively, the distinct modulation mechanisms of proton and small-molecule ligands in GPR4 and GPR65, could be particularly impactful in understanding the diverse physiological and pathological roles for proton-sensitive GPCRs, which could also provide new strategies to address inflammatory and pH sensing-related diseases.

## Materials and methods

### Bacterial strains

*Escherichia coli* strain DH5α was cultured at 37 °C in LB medium with ampicillin at a concentration of 100 μg/mL for extraction of plasmid DNA. For protein expression of Nanobody-35 (Nb35), *E. coli* strain BL21 (DE3) was cultured at 30 °C in LB medium with 100 μg/mL ampicillin, and induced during log phase growth with 0.5 mM isopropyl β-D-1-thiogalactopyranoside (IPTG) for 3 h at 25 °C.

### Cell line

*Super-Spodoptera frugiperda* insect cells (*ssf*9-1, *ssf*9-2, *ssf*9-3), along with the Bac-to-Bac baculovirus expression system (Invitrogen) were used to express different GPR4 and GPR65 complexes. Cell cultures were grown in ESF 921 serum-free medium (Expression Systems).

For the cell-based functional assays, human embryonic kidney 293T cells (HEK293T) cells were cultured in Dulbecco’s Modified Eagle Medium (DMEM, Corning) supplemented with 10% (v/v) fetal bovine serum (FBS, Gibco) at 37 °C in humidified air with 5% CO_2_.

### Construct generation for GPR4, GPR65 and G protein heterotrimers

For GPR4, the protein Endoglucanase H (PDB: 2CIT)^36^ was fused to the N-terminus of WT human GPR4, and the C-terminal residues (355–362) were truncated to improve the receptor expression and homogeneity. The modified construct was then subcloned into the pFastBac1 vector which contains a haemagglutinin signal peptide (HA) followed by a Flag tag at the N-terminus and an HRV-3C site followed by a His_10_-tag at the C-terminus. The dominant-negative human Gα_s_ (DNGα_s_)^37^ with 8 mutations including I285T, T284D, R280K, K274D, N271K, E268A, G226A, and S54N was used. The miniGα_13_ was obtained by introducing 7 stabilizing mutations: G57D^S1H1.03^, E58N^S1H1.04^, S248D^S4.07^, E251D^S4H3.03^, I271D^H3.08^, I355A^H5.04^ and V358I^H5.07^ along with replacing the α-helical domain with a GGSGGSGG linker.^38^ The miniGα_s/q_ was engineered with 7 stabilizing mutations R380K^H5.12^, Q384L^H5.16^, R385Q^H5.17^, H387N^H5.19^, Q390E^H5.22^, E392N^H5.24^, and L394V^H5.26^.^39^ These previously described Gα together with Gβ_1_γ_2_, were used for GPR4–G_s_, GPR4–miniG_13_ and GPR4–miniG_s/q_ complex formation, respectively.

For the inactive-state construct of human GPR4, a BRIL domain with a short linker derived from the A_2A_ receptor^40^ was inserted into the ICL3, replacing residues 211–216 of GPR4. To improve protein stability, the C-terminal residues 355–362 were truncated, and D63^2.50^N mutation was introduced to improve the protein yield and stability. The construct contained a 10× His tag and an HRV-3C site at the C-terminus. HA signal peptide, Flag tag and Endoglucanase H (PDB: 2CIT) were added on the N-terminus to enhance receptor expression. To improve the stability of the complex and reduce aggregation, the MBP protein was fused to the C-terminus of the anti-BRIL Fab heavy chain.

For GPR65, the modified N-terminal BRIL (PDB: 1M6T)^41^-fused WT human GPR65 construct was cloned into pFastBac1 vector, which includes a C-terminal His_6_-tag, HRV 3C site, and N-terminal HA signal peptide, Flag tag and tobacco etch virus (TEV) protease site. T234^6.43^I mutation was introduced to further enhance the surface expression of GPR65. miniGα_s_399^29^ and Gβ_1_γ_2_ were used for GPR65–miniG_s_ complex constitution.

### GPR4–G_s_–Nb35, GPR4–G_q_–Nb35 and GPR4–miniG_13_–scFv16 complex formation and purification

Using the Bac-to-Bac baculovirus expression system (Invitrogen), the modified GPR4 was co-expressed with DNGα_s_ or miniGα_13_, along with Gβ_1_γ_2_ in *ssf*9-2 insect cells, which were infected at a cell density of 2–2.5 × 10^6^ cells per milliliter with three separate virus preparations for GPR4, DNGα_s_ or miniGα_13_ and Gβ_1_γ_2_ at a ratio of 4:1:1. The infected cells were cultured at 27 °C for 48 h before collection by centrifugation and the cell pellets were stored at –80 °C for future use. For GPR4–miniG_s_ and GPR4–miniG_s/q_ complexes, protein expression was performed using *ssf*9-3 insect cells with the same transfection ratio employed for DNGα_s_ and miniGα_13_.

The purification procedures for _pH7.5_GPR4–G_s_, _pH6.8_GPR4–G_s_, _pH6.0_GPR4–G_s_ and _pH8.0_GPR4–G_s_ complexes were similar except for adjusting the buffer pH to 7.5, 6.8, 6.0 and 8.0, respectively. For _pH7.5_GPR4–G_s_ and _pH6.8_GPR4–G_s_, the cell pellets corresponding to 1 L GPR4–DNGα_s_ co-expression culture were thawed and lysed in the hypotonic buffer (50 mM HEPES, 2 mM MgCl_2_, 50 mM NaCl, 10 μg/mL Nb35, 25 mU/mL apyrase (NEB), and EDTA-free complete protease inhibitor cocktail tablets (Roche)). The lysate was incubated at 4 °C for 6 h and then the membranes were collected by centrifugation at 35,000 rpm for 35 min. The complex was displaced from the membranes using solubilization buffer (25 mM HEPES, 100 mM NaCl, 1% (w/v) LMNG, 0.2% (w/v) CHS) for 2.5 h at 4 °C. The supernatant was collected by centrifugation at 35,000 rpm for 50 min and incubated with TALON IMAC resin (Clontech) and 30 mM imidazole overnight at 4 °C. The resin was washed with 15 column volumes (CVs) of washing buffer I (25 mM HEPES, 100 mM NaCl, 10% (v/v) glycerol, 0.05% (w/v) LMNG, 0.01% (w/v) CHS, 30 mM imidazole), and 15 CVs of washing buffer II (25 mM HEPES, 100 mM NaCl, 10% (v/v) glycerol, 0.01% (w/v) LMNG, 0.002% CHS, 50 mM imidazole). The complex was eluted by 4 CVs of eluting buffer (25 mM HEPES, 100 mM NaCl, 10% (v/v) glycerol, 0.01% (w/v) LMNG, 0.002% CHS, 250 mM imidazole). The GPR4–DNG_s_–Nb35 complex was concentrated and injected onto a Superdex 200 Increase 10/300 column (GE Healthcare) equilibrated with buffer (20 mM HEPES, 100 mM NaCl, 0.00075% (w/v) LMNG, 0.0001% (w/v) CHS, 0.1 mM TCEP). The GPR4–DNG_s_–Nb35 complex peak fractions were collected and concentrated to 4.0–5.0 mg/mL for cryo-EM sample preparation. The purification procedures for _pH6.0_GPR4–G_s_ and _pH8.0_GPR4–G_s_ are similar to that of _pH7.5_GPR4–G_s_ and _pH6.8_GPR4–G_s_ complexes except that the buffer solution was replaced by MES or EPPS instead of HEPES.

For _pH6.8_GPR4–miniG_13_, the purification method is similar to that of _pH6.8_GPR4–G_s_ complex, in which scFv16^42^ was used to stabilize the complex instead of Nb35. For _pH7.5_GPR4–miniG_s/q_ and _pH6.8_GPR4–miniG_s/q_, the purification method is similar to that of _pH7.5_GPR4–G_s_ and _pH6.8_GPR4–G_s_ complexes.

### Purification of inactive GPR4

The inactive GPR4 was expressed in *ssf*9-3 insect cells, and cells were infected with a high-titer virus at 27 °C and collected 48 h after infection. For purification, cells were lysed and washed with low-salt buffer containing 10 mM HEPES, pH 7.5, 20 mM KCl, 10 mM MgCl_2_ and EDTA-free protease inhibitor cocktail (Roche), followed by washing with high-salt buffer containing 10 mM HEPES, pH 7.5, 20 mM KCl, 1 M NaCl, 10 mM MgCl_2_ and EDTA-free protease inhibitor cocktail. Then, 100 μM NE52-QQ57 (MCE, HY-101784) was added and incubated with purified membranes at 4 °C for 2 h, followed by addition of 2 mg/mL iodoacetamide for another 1 h. GPR4 protein was extracted with 1% (w/v) LMNG, 0.2% (w/v) CHS for 2.5 h at 4 °C. The supernatant was collected by ultracentrifugation at 35,000 rpm for 50 min and incubated with TALON IMAC resin and 20 mM imidazole at 4 °C overnight. The resin was washed with 20 CVs of wash buffer I containing 25 mM HEPES, pH 7.5, 100 mM NaCl, 10% (v/v) glycerol, 0.01% (w/v) LMNG and 0.002% (w/v) CHS, 30 mM imidazole and 100 μM NE52-QQ57. Then the resin was washed with 20 CVs of wash buffer II containing 25 mM HEPES, pH 7.5, 100 mM NaCl, 10% (v/v) glycerol, 0.01% LMNG and 0.002% CHS, 50 mM imidazole and 100 μM NE52-QQ57. The protein was further eluted with 25 mM HEPES, pH 7.5, 100 mM NaCl, 10% (v/v) glycerol, 0.01% (w/v) LMNG and 0.002% (w/v) CHS, 250 mM imidazole and 100 μM NE52-QQ57, and the imidazole was removed using a PD MiniTrap G-25 column (GE HealthCare). Purified GPR4 receptor was incubated with 1.5-fold and 2-fold molar excesses of MBP-anti-BRIL Fab and anti-Fab Nb, respectively. The mixture was incubated at 4 °C for 4 h. Then the complex was loaded on a Superdex 200 Increase 10/300 column in AKTA buffer (20 mM HEPES, pH 7.5, 100 mM NaCl, 0.00075% (w/v) LMNG, 0.0001% (w/v) CHS, and 100 µM NE52-QQ57). Peak fractions containing complexes were concentrated to 20 mg/mL for cryo-EM study.

### GPR65–miniG_s_–Nb35 complex formation and purification

The modified GPR65 was co-expressed with miniGα_s399_, Gβ_1_γ_2_ in *ssf*9-3 insect cells, which were infected at a cell density of 2.0–2.5 × 10^6^ cells per milliliter with three separate virus preparations for GPR65, miniGα_s399_ and Gβ_1_γ_2_ at a ratio of 3:1:1. The infected cells were collected by centrifugation after 48 h and the cell pellets were stored at –80 °C.

The *ssf*9-3 insect cell pellets of the 1.0 L co-expression culture (GPR65–miniG_s_) were thawed and lysed in binding buffer (10 mM MES, 10 mM MgCl_2_, 20 mM KCl, 10 mM CaCl_2_, 0.1 mM TCEP, 400 μg Nb35, 25 mU/mL apyrase and EDTA-free complete protease inhibitor cocktail tablets (Roche)). The lysate was incubated at 4 °C for 6 h and then the membranes were collected by centrifugation at 35,000 rpm for 35 min. The complex was isolated from the membranes using the solubilization buffer (20 mM MES, 100 mM NaCl, 0.75% (w/v) LMNG, 0.15% (w/v) CHS, 10 mM CaCl_2_, 0.1 mM TCEP) for 2.5 h at 4 °C. The supernatant was collected by centrifugation at 35,000 rpm for 50 min and incubated with TALON IMAC resin and 20 mM imidazole overnight at 4 °C. The resin was washed with 20 CVs of washing buffer I (20 mM MES, 100 mM NaCl, 10% (v/v) glycerol, 0.03% (w/v) LMNG, 0.006% (w/v) CHS, 30 mM imidazole), and 20 CVs of washing buffer II (20 mM MES, 100 mM NaCl, 10% (v/v) glycerol, 0.01% (w/v) LMNG, 0.002% CHS, 50 mM imidazole). The complex was eluted by 4 CVs of eluting buffer (20 mM MES, 100 mM NaCl, 10% (v/v) glycerol, 0.01% (w/v) LMNG, 0.002% CHS, 250 mM imidazole). The GPR65–miniG_s_–Nb35 complex was concentrated to 1 mL and injected onto a Superdex 200 Increase 10/300 column equilibrated with buffer (20 mM HEPES, 100 mM NaCl, 0.00075% (w/v) LMNG, 0.0001% (w/v) CHS, 0.1 mM TCEP). The complex peak fractions were collected and concentrated to 5.0–6.0 mg/mL for cryo-EM sample preparation.

### Cryo-EM grid preparation and data collection

A 3.0 μL aliquot of concentrated complexes (GPR4–G_s_, GPR4–miniG_13_, GPR4–miniG_s/q_, GPR65–miniG_s_ or GPR4–Fab–Nb) was applied onto glow-discharged holey carbon grids. Prior to sample application, a controlled glow-discharge process was applied to the EM grids, utilizing a mixture of hydrogen (H_2_) and oxygen (O_2_) gases for 40 s. The vitrification procedure was performed within the FEI Vitrobot Mark IV chamber (Thermo Fisher Scientific), where the humidity was elevated to 100% while maintaining a temperature of 4 °C.

For GPR4–G_s_ and GPR65–miniG_s_ complexes, the subsequent cryo-EM images were collected using a Titan Krios microscope operating at 300 kV, equipped with a Gatan K3 summit direct electron camera. Each movie was recorded with EFTEM nanoprobe mode, employing a 70-μm C2 aperture, while adhering to a calibrated magnification of 105,000, resulting in an amplified pixel size of 0.832 Å. Each movie consisted of 40 frames, and a cumulative electron dose of 60 electrons per Å^2^ was delivered. The data acquisition was executed through the utilization of SerialEM software,^43^ employing a defocus range from –1.2 μm to –2.0 μm.

For GPR4–miniG_13_, GPR4–miniG_s/q_ and NE52-QQ57–GPR4–Fab–Nb complexes, the subsequent cryo-EM images were collected using a Titan Krios microscope operating at 300 kV, equipped with the Falcon 4 Direct Electron Detector (Thermo Fisher Scientific). Each movie was recorded at a calibrated magnification of 130,000, resulting in an amplified pixel size of 0.96 Å. Each movie consisted of 40 frames, and a cumulative electron dose of 60 electrons per Å^2^ was delivered. The data acquisition was executed through the utilization of EPU software, employing a defocus range from –1.2 μm to –2.0 μm for GPR4–miniG_13_ and _pH7.5_GPR4–miniG_s/q_ complexes, and –0.8 μm to –1.5 μm for _pH6.8_GPR4–miniG_s/q_ and NE52-QQ57–GPR4–Fab–Nb complexes.

### Cryo-EM data processing

For the _pH7.5_GPR4–DNG_s_–Nb35, _pH6.8_GPR4–DNG_s_–Nb35, _pH6.0_GPR4–DNG_s_–Nb35, _pH8.0_GPR4–miniG_s_–Nb35, _pH6.8_GPR4-–miniG_13_–scFv16, _pH7.5_GPR4–miniG_s/q_–Nb35, _pH6.8_GPR4–miniG_s/q_–Nb35, NE52-QQ57–GPR4–Fab–Nb and _pH6.5_GPR65–miniG_s_–Nb35 complexes, we collected a total of 9348, 8960, 8595, 7329, 7102, 13,927, 6311, 6056 and 11,939 movie stacks, respectively. To address beam-induced motion, we applied MotionCor 2.1^44^ for motion correction across all movie stacks. Subsequently, we utilized cryoSPARC v4.3.0^45^ to employ patch contrast transfer function estimation, thereby deriving the contrast transfer function parameters for each micrograph.

The complexes, specifically _pH7.5_GPR4–DNG_s_–Nb35, _pH6.8_GPR4–DNG_s_–Nb35, _pH6.0_GPR4–DNG_s_–Nb35, _pH8.0_GPR4–miniG_s_–Nb35 and _pH6.8_GPR4–miniG_13_–scFv16, yielded a total of 2,937,152, 2,366,130, 2,381,207, 3,035,278 and 1,092,391 particle projections, respectively. In order to eliminate false-positive particles, a series of iterative two- and three-dimensional categorizations were applied to all particles in cryoSPARC. This refinement process led to the generation of final datasets that included 374,926, 314,168, 157,993, 215,671 and 171,879 particle projections from the most optimal classes. These datasets were then subjected to subsequent processing stages involving final homogenous refinement, and non-uniform refinement in cryoSPARC. Primary models were constructed based on the map generated from non-uniform refinement. To mitigate the influence of micelles on the final map, volumes at a resolution of 24 Å were generated from the primary models using the ‘molmap’ command in Chimera. These volumes were then imported into CryoSPARC, where a mask was created using the Volume Tools feature with a threshold set to 0.12 and a dilation radius of 10 pixels. Subsequently, a local refinement job was performed, followed by local sharpening using DeepEMhancer. As a result of these steps, density maps were successfully generated with nominal resolutions of 2.9 Å, 3.1 Å, 3.1 Å, 2.8 Å and 3.4 Å (determined by Fourier shell correlation (FSC) using the 0.143 criterion) for the _pH7.5_GPR4–DNG_s_–Nb35, _pH6.8_GPR4–DNG_s_–Nb35, _pH6.0_GPR4–DNG_s_–Nb35, _pH8.0_GPR4–miniG_s_–Nb35 and _pH6.8_GPR4–miniG_13_–scFv16 complexes, respectively.

For the NE52-QQ57–GPR4–Fab–Nb complex, automated particle selection using Topaz extract in CryoSPARC produced 2,569,899 particles. The particles were imported into CryoSPARC for several rounds of 2D classification and ab initio reconstruction to generate the initial reference maps, followed by three rounds of heterogeneous refinement in CryoSPARC. The good particles accounting for 151,171 particles were subjected to homogeneous refinement and non-uniform refinement. A primary model was constructed based on the map generated from non-uniform refinement. To mitigate the influence of micelles on the final map, a volume at the resolution of 24 Å was generated from the primary model using the ‘molmap’ command in Chimera. This volume was then imported into CryoSPARC, where a mask was created using the Volume Tools feature with a threshold set to 0.08 and a dilation radius of 2 pixels. Subsequently, a local refinement job was performed, followed by local sharpening using DeepEMhancer. As a result of these steps, a map was successfully generated with an indicated global resolution of 3.2 Å in CryoSPARC.

For the _pH7.5_GPR4–miniG_s/q_–Nb35 complex, the good particles accounting for 1,306,046 particles were subjected to homogeneous refinement and non-uniform refinement. A primary model was constructed based on the map generated from non-uniform refinement. To mitigate the influence of micelles on the final map, a volume at the resolution of 24 Å was generated from the primary model using the ‘molmap’ command in Chimera. This volume was then imported into CryoSPARC, where a mask was created using the Volume Tools feature with a threshold set to 0.12 and a dilation radius of 10 pixels. Subsequently, a local refinement job was performed, generating a map. The map and particles from local refinement were used for 3D classification, resulting in 10 output classes. Two classes with well-defined structural details underwent further local refinement using masks generated from the 3D classification, yielding two global maps, state-1 (142,072 particles) and state-2 without ligand (137,792 particles), at a resolution of 2.9 Å (FSC 0.143).

For the _pH6.8_GPR4–miniG_s/q_–Nb35 complex, 6311 video stacks were imported into CryoSPARC v4.3. A total of 3,631,608 particles were extracted and then subjected to reference-free 2D classification to remove the bad particles. The picked particle set was subjected to ab initio reconstruction and heterogeneous refinement served as 3D classification, generating four subclasses. Then 276,423 particle projections of the best class were further applied for final homogenous refinement and non-uniform refinement. A primary model was constructed based on the map generated from non-uniform refinement. To mitigate the influence of micelles on the final map, a volume at the resolution of 24 Å was generated from the primary model using the ‘molmap’ command in Chimera. This volume was then imported into CryoSPARC, where a mask was created using the Volume Tools feature with a threshold set to 0.08 and a dilation radius of 2 pixels. Subsequently, a local refinement job was performed, followed by local sharpening using DeepEMhancer, yielding the final density map with a global resolution of 2.7 Å.

For the _pH6.0_GPR65–miniG_s_–Nb35 complex, 11,939 movie stacks were collected. Subsequently, we extracted 379,721 particles for 3D heterogeneous refinement. Following this, we selected 80,542 particle projections representing the best class for further refinement procedures. This involved a combination of homogeneous refinement, and non-uniform refinement within cryoSPARC. A primary model was constructed based on the map generated from non-uniform refinement. To mitigate the influence of micelles on the final map, a volume at the resolution of 24 Å was generated from the primary model using the ‘molmap’ command in Chimera. This volume was then imported into CryoSPARC, where a mask was created using the Volume Tools feature with a threshold set to 0.08 and a dilation radius of 2 pixels. Subsequently, a local refinement job was performed, followed by local sharpening using DeepEMhancer, resulting in the final density map at a resolution of 3.3 Å.

### Model building and refinement

We used coordinates of GPR4 and GPR65 predicted by AlphaFold2 as the initial models. The DNG_s_, miniGα_s399_, miniG_s/q_ and Nb35 were generated using the GPR139–JNJ-63533054–miniG_s/q_–Nb35 complex^39^ (PDB: 7VUJ). The miniG_13_ heterotrimer (miniGα_13_, Gβ1 and Gγ2) and scFv16 were generated using the GPR139–JNJ-63533054–G_i_ complex (PDB: 7VUG) as the initial models. The anti-BRIL Fab and anti-Fab Nb was generated using the FZD3 in apo inactive-state complex^46^ (PDB: 8JHC). The cryo-EM model was fit into the density map using Chimera.^47^ Subsequent refinement iterations involved manual adjustments and reconstruction in COOT,^48^ followed by phenix.real_space_refine in Phenix.^49^ To ensure the quality of the model, its statistical characteristics were assessed using MolProbity.^50^ Structural figures were generated using ChimeraX^51^ and PyMoL (http://www.pymol.org). The comprehensive refinement statistics can be found in Supplementary information, Table S1.

### Cell culture

HEK293T cells were procured from the American Type Culture Collection (ATCC). The cells were cultured and propagated in DMEM medium (Corning, New York, USA), supplemented with 10% (v/v) FBS (Gibco-Thermo Fisher Scientific, Waltham, USA), 100 U/mL penicillin, 1 mM sodium pyruvate (Gibco-Thermo Fisher Scientific), and 100 μg/mL streptomycin (Gibco-Thermo Fisher Scientific). These culture conditions were maintained at a temperature of 37 °C with a 5% CO_2_ atmosphere. For subsequent functional experiments, transient transfection of GPR4 or GPR65 was conducted.

### cAMP accumulation assay

The accumulation of cAMP upon proton activation was quantified using the Glosensor^TM^ cAMP assay (Promega). The WT forms of GPR4 and GPR65, mutants, and N-2CIT construct were subcloned into the pcDNA3.1 vector, featuring the N-terminal HA and Flag tags. Briefly, HEK293T cells were cultured in 6-well plates and then transfected when reaching a confluency of 70%–80%. Transfection involved introducing 500 ng of the GPR65 WT, GPR4 WT, mutant, or N-2CIT construct along with 1250 ng of the pGloSensor-22F cAMP plasmids (Promega) into the cells. Subsequently, the transfected cells were seeded into 384-well white clear-bottom cell culture plates coated with poly-L-lys at a density of 15,000 cells per well in 40 μL of DMEM supplemented with 1% dialyzed FBS. After 12 h of culture, the cells were exposed to a D-luciferase solution (2 mg/mL in substrate buffer) for 90 min at 37 °C. The substrate buffer was prepared with 1× Hank’s balanced salt solution (HBSS) containing 20 mM EPSS at pH 8.5. Furthermore, assay buffers at various pH levels were prepared at 25 °C for GPR4 (pH = 8.5, 8.0, 7.8, 7.6, 7.5, 7.4, 7.2, 7.0, 6.8, 6.6, 6.5, 6.2, 6.0, 5.8, 5.5, 5.0) and GPR65 (pH = 8.5, 8.0, 7.5, 7.2, 7.0, 6.8, 6.6, 6.5, 6.4, 6.2, 6.0, 5.8, 5.5, 5.3, 5.0, 4.5). These buffers were composed of 20 mM EPPS in 1× HBSS solution, 20 mM HEPES in 1× HBSS solution, or 20 mM MES in 1× HBSS solution, corresponding to different pH ranges. The pH levels were adjusted using NaOH and HCl and were measured at either 25 °C or 37 °C. Following the removal of the D-luciferase solution, 30 μL of assay buffer was added to each well, and the cells were equilibrated at 25 °C for 40 min (for GPR4) or 30 min (for GPR65) before readings were taken using an Envision multilabel plate reader (Perkin Elmer).

For antagonist NE52-QQ57, cells were incubated with D-luciferase solution (2 mg/mL prepared in drug buffer, pH 7.5) for 2 h at room temperature. The drug buffer was prepared with 20 mM HEPES and 1× HBSS. Then 10 μL drug buffer with 3× concentrated compound was added to cells and incubated at room temperature for 1 h before being counted in an Envision multilabel plate reader (Perkin Elmer).

### Intracellular calcium mobilization assay

CHO-K1 cells (ATCC) were cultured in F-12 medium, supplemented with 10% FBS and 1% penicillin/streptomycin. When the cells reached ~70% confluence in a 6-well plate, they were transfected with 1 µg of either GPR4 or its mutant DNA using TransIT 2020 (Mirus Bio). After 24 h, the cells were trypsinized and seeded into black, clear-bottom 384-well cell culture plates (Greiner) at a density of 10,000 to 15,000 cells per well in 40 µL of growth medium. The following day, the growth medium was removed, and the cells were loaded with 20 µL per well of 1× Fluo-4 Direct Calcium dye (prepared in HBSS buffer, pH 8.0) (Invitrogen). The plates were incubated at 37 °C for 1 h in the dark. The FLIPR system was programmed to capture 10 baseline fluorescence readings (1 read per second) prior to the addition of 10 µL of serial pH solutions. Serial pH solutions, ranging from 5.0 to 8.5, were prepared using different buffer agents: 20 mM EPPS, HEPES, or MES in 1× HBSS, tailored to specific pH ranges. The pH adjustments were performed with NaOH and HCl, and measurements were taken at 25 °C. Following the addition of test solutions, fluorescence intensity was recorded for 2 min. Data analysis was conducted using nonlinear regression with GraphPad Prism 10.

### Surface expression analysis

Cell-surface expression for WT and mutants of GPR4 and GPR65 was monitored by fluorescence-activated cell sorting assay. HEK293T cells were initially seeded into 96-well plates at a density of 15,000 cells per well and transfected after 20 h. After 48 h post-transfection, the cells were detached using versene solution, followed by treatment with 100 µL per well of Dulbecco’s Phosphate Buffered Saline (DPBS) containing 10% FBS. Subsequently, the cells were incubated with 20 µL per well of the mouse anti-Flag (M2-phycoerythrin (PE)) antibody (Abcam) for 12 h at 4 °C. Following the incubation with the anti-Flag antibody, the cells were diluted with 100 µL of DPBS and subjected to flow cytometry analysis using a Guava flow cytometer (Millipore, Burlington, USA).

### G protein dissociation assays

G protein BRET probes, including Gα_13_–RLuc, Gβ and Gγ–GFP were generated, and the Gα_q_–Gβγ dissociation assay was performed as previously described.^52^ HEK293T cells were transiently cotransfected with WT GPR4 and G protein BRET probes. HEK293T cells were distributed into 96-well microplates at a density of 5 × 10^4^ cells per well and incubated for another 24 h at 37 °C after 24 h transfection. The cells were washed once with assay buffer, 1× HBSS containing 20 mM HEPES, and then stimulated with the same assay buffer used in the cAMP accumulation assay at different pH levels. BRET signals were measured after addition of the luciferase substrate coelenterazine 400a (5 μM) using a Tristar 5 LB942 multimode plate reader (Berthold Technologies, Bad Wildbad, Germany) with BRET filter sets, and the signal was collected at integration times of 1 s for each well. The BRET signal was calculated as the ratio of light emission at 515 nm/410 nm.

### Calculation of the protonation states of key histidine residues

In accordance with methods from previous studies, the p*K*_a_ values were determined using the H++ server,^53,54^ calculated as pK_(1/2), which is equivalent to p*K*_a_ in most cases. For all p*K*_a_ calculations, the pH values were set to match those used in the experimental conditions. Additionally, the proportion of protonated species was determined using the Henderson-Hasselbalch equation.^55^

### Molecular docking

Molecular docking was performed using Schrödinger software to assist the NE52-QQ57 modeling in the inactive structure of GPR4. First, the receptor portion of GPR4 was prepared under the OPLS4 force field^56^ by adding hydrogens, optimizing the hydrogen bond assignment, and performing energy minimization. The ligand preparation for the NE52-QQ57 molecule was carried out using LigPrep. For the standard docking described in the manuscript, the ‘Glide’^57^ SP precision was used for sampling. Additionally, the docking of small molecule poses based on cryo-EM densities was performed using GemSpot.^58^ We employed the tool’s default protocol and provided the residue numbers of the surrounding region for sampling.

### MD simulations and analysis

For the GPR4 complex, the receptor portion was retained for the system construction. The protonation states of residues at different pH values were obtained from the previously mentioned website. The protein was inserted into a lipid bilayer composed of 150 1-palmitoyl-2-oleoyl-sn-glycero-3-phosphatidylcholine molecules using CHARMM-GUI,^59^ with water molecules modeled using the TIP3P model. Additionally, 0.15 M sodium and chloride ions were added to neutralize the system’s charge. MD simulations were performed using GROMACS 2021 software, employing the CHARMM36m force field^60^ with a cutoff distance of 12 Å for nonbonded interactions and the Particle Mesh Ewald method^61^ for long-range van der Waals interactions. Energy minimization was performed using the default bilayer protein protocol in CHARMM-GUI. The system was then equilibrated under the NVT ensemble, gradually heating from 0 K to 310 K over 150 ps. Following this, the system was equilibrated under the NPT ensemble at 310 K and 1 bar, during which the restraints on the protein and lipid bilayer were gradually released. Finally, a 200 ns production phase was performed, where all heavy atoms, except for those in the D161 and H165 residues, were subjected to a restraint potential of 50 kJ/mol·nm². Analysis of the MD trajectory was conducted using the distance module provided by GROMACS.

### Identification of lipids associated with GPR4–G_q_ by liquid chromatography-tandem mass spectrometry (LC-MS/MS)

Endogenous lipids bound to GPR4–G_q_ complex were identified as previously described.^62,63^ Briefly, NE52-QQ57–GPR4–Anti-Bril Fab and _pH7.5_GPR4–G_q_ complexes were separately reduced with 5 mM TCEP and alkylated with 20 mM iodoacetamide at 25 °C. Then ~25 μg protein samples were digested with trypsin (Promega, Madison, USA) at an enzyme-to-protein ratio of 1:50 (w/w) at 37 °C for 12–14 h. The protein digests were dried in a SpeedVac machine and then extracted with ice-cold methanol:water (9:1, v/v) by vortex and sonication. The supernatants were collected to lyophilize after centrifugation at 16,000× *g* for 20 min at 4 °C. The lipid extracts were resuspended in methanol:chloroform (9:1, v/v) to an equivalent concentration of 2 μM. Samples were analyzed on a Q Exactive HF mass spectrometer (Thermo Fisher Scientific) operating in the positive ion mode coupled to a Waters Acquity UPLC system (Waters). The LC separation was performed on a CSH C18 column (10 mm × 2.1 mm; 1.7 μm) (Waters, Milford, MA) at a flow rate of 0.3 mL/min at 40 °C, with the mobile phase A consisting of acetonitrile:water (60:40, v/v) with 10 mM ammonium formate and 0.1% formic acid, and B consisting of 2-propanol:acetonitrile (90:10, v/v) with 10 mM ammonium formate and 0.1% formic acid. The LC gradient was set as follows: 0 min 15% B; 0–4 min 30% B; 4–4.5 min 48% B; 4.5–22 min 82% B; 22–24 min 99% B. MS data acquisition was set to the following parameters: mass range 160–1500 *m*/*z*; spray voltage 3.5 kV; sheath gas (nitrogen) flow rate 35 units; auxiliary gas (nitrogen) flow rate 10 units; capillary temperature 320 °C. MS1 scan parameters included resolution 60,000, AGC target 3e6, and maximum injection time 250 ms. MS/MS spectra were acquired on the top 10 precursors with normalized collision energy set at 10 eV, 20 eV and 40 eV. All samples were prepared in three independent replicates.

The LC-MS/MS data for lipid extracts from NE52-QQ57–GPR4–Anti-Bril Fab and _pH7.5_GPR4–G_q_ complexes were processed with MS-DIAL (v4.90). For lipid identification, the accurate mass and tandem mass spectra were matched with a built-in lipid spectral library LipidBlast,^64^ and their retention times were aligned with available reference compounds. MS spectra for all identified lipids were also manually inspected and confirmed. The quantitative MS responses of identified lipids, defined as the peak areas of extracted ion chromatograms, were extracted from LC-MS/MS data using Xcalibur (v4.0, Thermo Fisher Scientific) based on accurate mass matching (< 3 ppm deviation) and retention time alignment with reference compounds. Student’s two-tailed *t*-test analysis was performed to determine statistical significance of the difference in MS responses of specific lipids associated with _pH7.5_GPR4–G_q_ vs NE52-QQ57–GPR4–Anti-Bril Fab (ns, no significance; ***P < 0.001, *n* = 4).

### Statistical analysis

Data analysis was conducted using Prism 9 software. In each experimental trial, the levels of proton-activated cAMP accumulation were assessed based on the fluorescence signals obtained from a minimum of 3 replicates. These values were normalized against the WT maximal signal, which was assigned a value of 100%. Comparative statistical analyses between the experimental values of the WT and mutant conditions were performed through one-way analysis of variance (ANOVA), followed by Dunnett’s post hoc test within GraphPad Prism 9.

## Supplementary information

Supplementary information, Figure S1

Supplementary information, Figure S2

Supplementary information, Figure S3

Supplementary information, Figure S4

Supplementary information, Figure S5

Supplementary information, Figure S6

Supplementary information, Figure S7

Supplementary information, Table S1

Supplementary information, Table S2

Supplementary information, Video S1

Supplementary information, Video S2

Supplementary information, Video S3

Supplementary information, Video S4

Supplementary information, Video legend

## Data Availability

The atomic coordinates for _pH8.0_GPR4–DNG_s_–Nb35, _pH7.5_GPR4–DNG_s_–Nb35, _pH6.8_GPR4–DNG_s_–Nb35, _pH6.0_GPR4–DNG_s_–Nb35, _pH6.8_GPR4–miniG_13_–scFv16, _pH7.5_GPR4–miniG_s/q_–state-1, _pH7.5_GPR4–miniG_s/q_-state-2, _pH6.8_GPR4–miniG_s/q_ and NE52-QQ57–GPR4–Fab complexes have been deposited in the Protein Data Bank (PDB) with the accession codes 9LGM, 8ZCF, 9JFW, 8ZCE, 9JHP, 9JFX, 9JFZ, 9JFV and 9JFU, respectively. The EM maps for _pH8.0_GPR4–DNG_s_–Nb35, _pH7.5_GPR4–DNG_s_–Nb35, _pH6.8_GPR4–DNG_s_–Nb35, _pH6.0_GPR4–DNG_s_–Nb35, _pH6.8_GPR4–miniG_13_–scFv16, _pH7.5_GPR4–miniG_s/q_-state-1, _pH7.5_GPR4–miniG_s/q_-state-2, _pH6.8_GPR4–miniG_s/q_ and NE52-QQ57–GPR4–Fab complexes have been deposited in the Electron Microscopy Data Bank (EMDB) with the codes EMD-63068, EMD-39928, EMD-61442, EMD-39927, EMD-61489, EMD-61443, EMD-61445, EMD-61441 and EMD-61440, respectively. The atomic coordinate for the _pH6.5_GPR65–miniG_s_–Nb35 complex has been deposited in the PDB with the accession code 9JFT. The EM map for the _pH6.5_GPR65–miniG_s_–Nb35 complex has been deposited in the EMDB with the code EMD-61439.
