# Supplementary material for: Structural basis of stepwise proton sensing-mediated GPCR activation
Source: Cell Res. 2025 Apr 11;35(6):423–36. doi: 10.1038/s41422-025-01092-w (PMC12134361; doi:10.1038/s41422-025-01092-w)
Supplement: Supplementary file 1 — Supplementary information, Figure S1 [file 41422_2025_1092_MOESM1_ESM.pdf]

Supplementary information, Figure S1

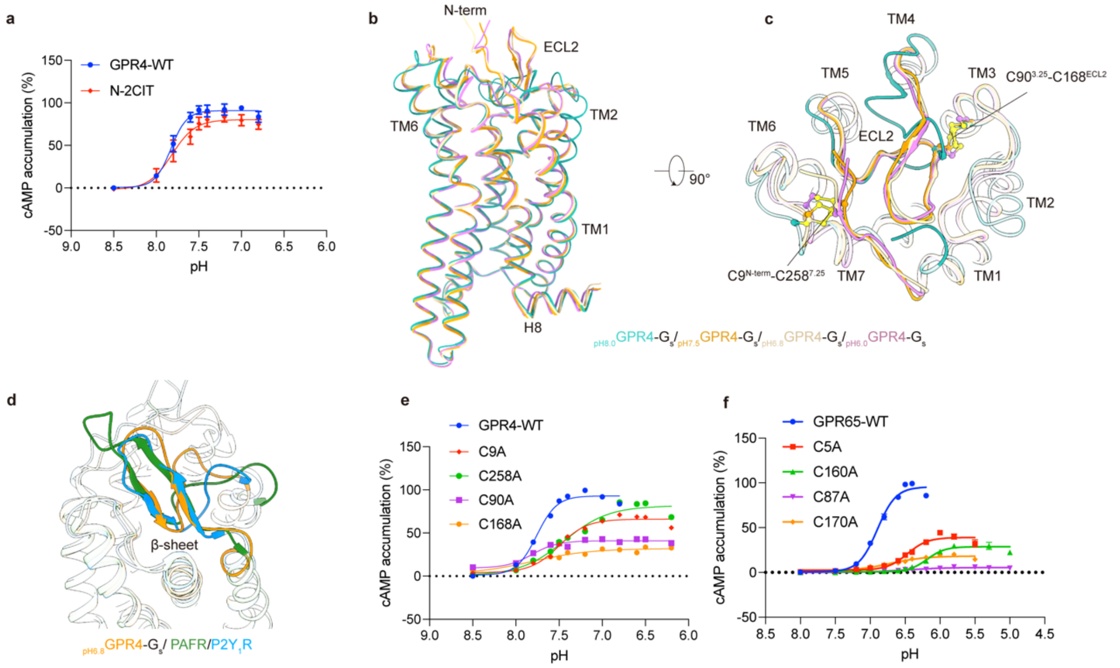

Fig. S1 Structures of different pH activated GPR4-Gs complexes. a, Concentration–response curves of protons with Fusion-GPR4 by measuring the cellular cAMP accumulation through the GloSensor assay. Data are means ± SEM of three biological replicates. **b-c,** Structural alignment of GPR4-G_s at pH7.5, pH6.8, pH6.0, and pH8.0 in side view (**b**) and extracellular view (**c**). **d,** ECL2 adopts the β-sheet hairpin structure in the ${}_{pH6.8}$GPR4–G_s complex, which is similar to the δ-branch GPCRs, PAFR (PDB: 5ZKP) and P2Y$_1$R (PDB: 7XXH). **e-f,** Concentration–response curves of protons with alanine mutations of residues forming disulfide bonds in GPR4 (**e**) and GPR65 (**f**) by measuring the cellular cAMP accumulation through the GloSensor assay. Data are means ± SEM of three biological replicates.
