# Supplementary material for: Structural basis of stepwise proton sensing-mediated GPCR activation
Source: Cell Res. 2025 Apr 11;35(6):423–36. doi: 10.1038/s41422-025-01092-w (PMC12134361; doi:10.1038/s41422-025-01092-w)
Supplement: Supplementary file 2 — Supplementary information, Figure S2 [file 41422_2025_1092_MOESM2_ESM.pdf]

Supplementary information, Figure S2

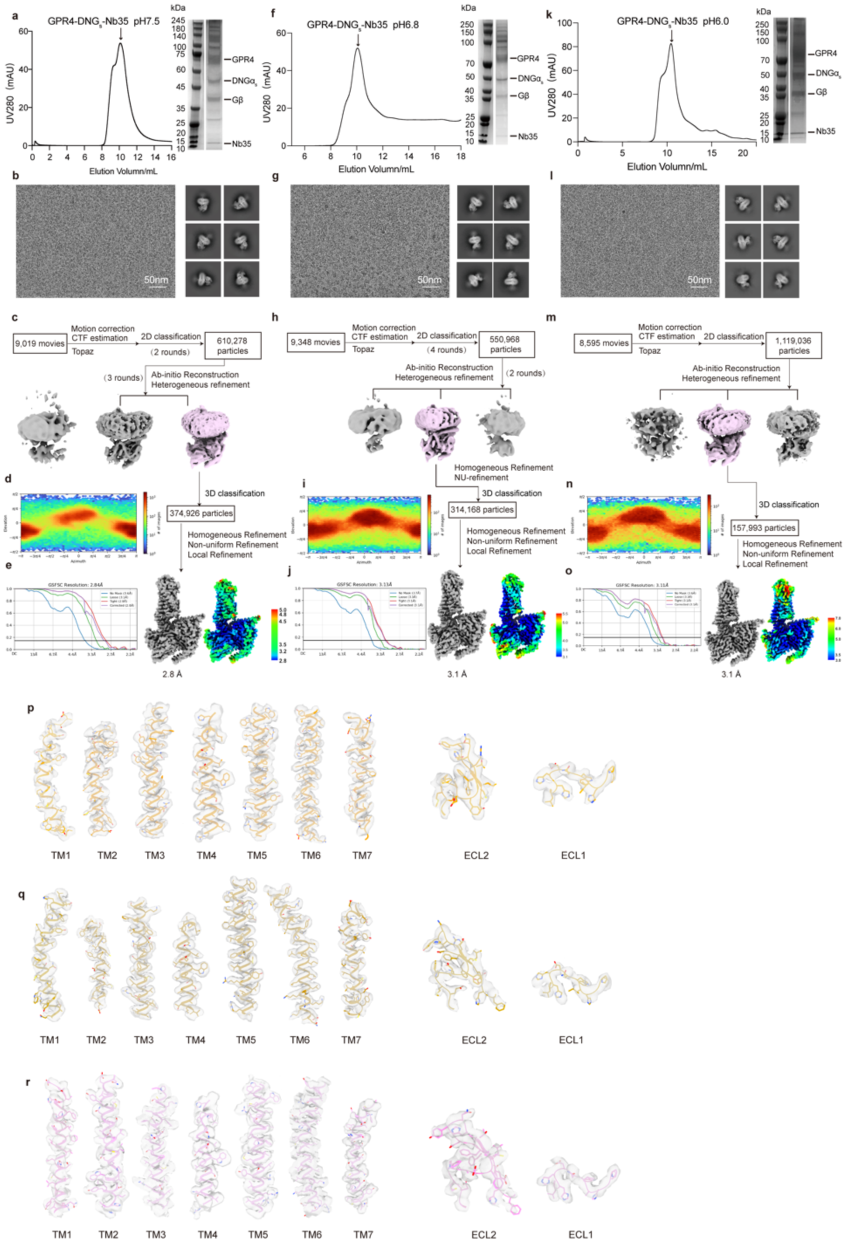

Fig. S2 Sample preparation and cryo-EM data processing of $_{pH7.5}$GPR4-DNG$_s$-Nb35, $_{pH6.8}$GPR4-DNG$_s$-Nb35 and $_{pH6.0}$GPR4-DNG$_s$-Nb35 complexes. a, f, k, Superdex200 size-exclusion chromatography elution profiles of the purified complex samples and SDS-PAGE analysis. **b, g, l,** Representative cryo-EM micrography and selected 2D classification of GPR4-G$_s$ complexes. **c, h, m,** Schematic representation of cryo-EM data processing workflow. **d, i, n,** Angular distribution of the particles used for final reconstruction. **e, j, o,** Cryo-EM maps are colored by local resolution (Å). The Fourier shell correlation (FSC) curves of GPR4-G$_s$. The global resolution of the final processed density map estimated at the FSC = 0.143. **p, q, r,** CryoEM maps and models

of TMs and ECLs for $_{pH7.5}$GPR4-DNG$_s$-Nb35 (**p**), $_{pH6.8}$GPR4-DNG$_s$-Nb35 (**q**) and $_{pH6.0}$GPR4-DNG$_s$-Nb35 (**r**) complexes, respectively.
