# Supplementary material for: Structural basis of stepwise proton sensing-mediated GPCR activation
Source: Cell Res. 2025 Apr 11;35(6):423–36. doi: 10.1038/s41422-025-01092-w (PMC12134361; doi:10.1038/s41422-025-01092-w)
Supplement: Supplementary file 3 — Supplementary information, Figure S3 [file 41422_2025_1092_MOESM3_ESM.pdf]

Supplementary information, Figure S3

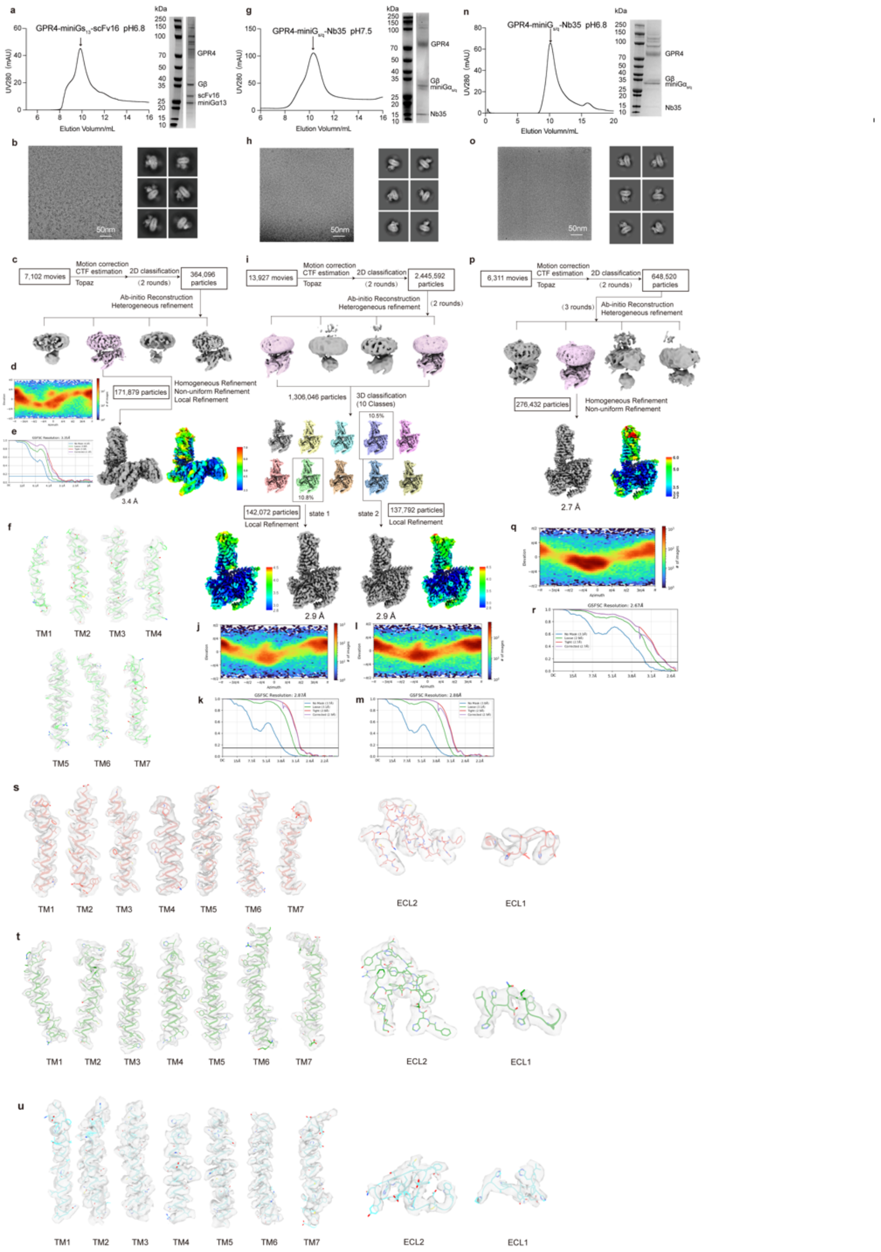

Fig. S3 Sample preparation and cryo-EM data processing of $_{\text{pH6.8}}$GPR4-miniG$_{13}$-scFv16, $_{\text{pH7.5}}$GPR4-miniG$_{s/q}$-Nb35 and $_{\text{pH6.8}}$GPR4-miniG$_{s/q}$-Nb35 complexes. a, g, n, Superdex200 size-exclusion chromatography elution profiles of the purified complex samples and SDS-PAGE analysis. **b, h, o,** Representative cryo-EM micrography and selected 2D classification. **c, i, p,** Schematic representation of cryo-EM data processing workflow. **d-e, j-m, q-r,** Angular distribution of the particles used for final reconstruction**,** cryo-EM maps are colored by local resolution (Å). The Fourier shell correlation (FSC) curves of $_{\text{pH6.8}}$GPR4-G$_{13}$, $_{\text{pH7.5}}$GPR4-G$_{q}$ and $_{\text{pH6.8}}$GPR4-G$_{q}$. The global resolution of the final processed density map estimated at the FSC = 0.143. **f,**

Cryo-EM maps and models of TMs for $_{pH6.8}$GPR4-miniG$_{13}$-scFv16. **s-u,** Cryo-EM maps and models of TMs and ECLs for $_{pH7.5}$GPR4-miniG$_{s/q}$-Nb35-state-1 (**s**), $_{pH7.5}$GPR4-miniG$_{s/q}$-Nb35-state-1 (**t**) and $_{pH6.8}$GPR4-miniG$_{s/q}$-Nb35 (**u**) complexes, respectively.
