# Supplementary material for: Structural basis of stepwise proton sensing-mediated GPCR activation
Source: Cell Res. 2025 Apr 11;35(6):423–36. doi: 10.1038/s41422-025-01092-w (PMC12134361; doi:10.1038/s41422-025-01092-w)
Supplement: Supplementary file 4 — Supplementary information, Figure S4 [file 41422_2025_1092_MOESM4_ESM.pdf]

**Supplementary information, Figure S4**

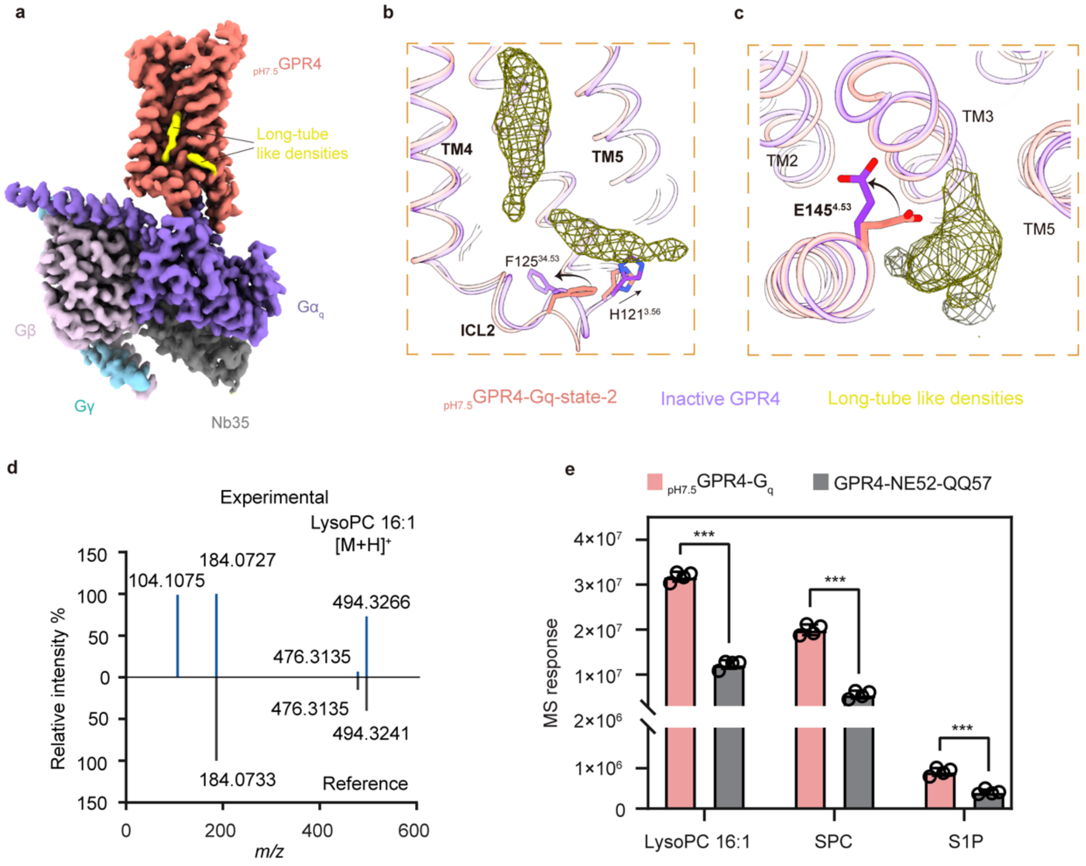


**Fig. S4 The lipids density map in $_{pH7.5}$GPR4-G$_q$ structure. a,** Two pieces of extra density were observed in GPR4-G$_q$ density map. **b,** The conformational changes of H121$^{3.56}$ and F125$^{34.53}$ in ICL2 between $_{pH7.5}$GPR4-G$_q$-state-2 and inactive GPR4 structures. c, The conformational change of E145$^{4.53}$ between $_{pH7.5}$GPR4-G$_q$-state-2 and inactive GPR4 structures. **d,** High-resolution MS/MS spectrum of LysoPC (16:1) identified from the $_{pH7.5}$GPR4-G$_q$ complex sample (upper, blue) aligned with the MS/MS spectrum of the reference compound recorded in LipidBlast database (lower, gray). **e,** MS responses of LysoPC (16:1), sphingosylphosphorylcholine (SPC) and sphingosine-1-phosphate (S1P) identified in GPR4-NE52-QQ57 and $_{pH7.5}$GPR4-G$_q$ complex samples. Data are presented as means ± SD (n=4 independent experiments). Student’s two-tailed t-test was performed to determine statistical significance (ns, no significance; ****P* <0.001).
