# Supplementary material for: Structural basis of stepwise proton sensing-mediated GPCR activation
Source: Cell Res. 2025 Apr 11;35(6):423–36. doi: 10.1038/s41422-025-01092-w (PMC12134361; doi:10.1038/s41422-025-01092-w)
Supplement: Supplementary file 5 — Supplementary information, Figure S5 [file 41422_2025_1092_MOESM5_ESM.pdf]

Supplementary information, Figure S5

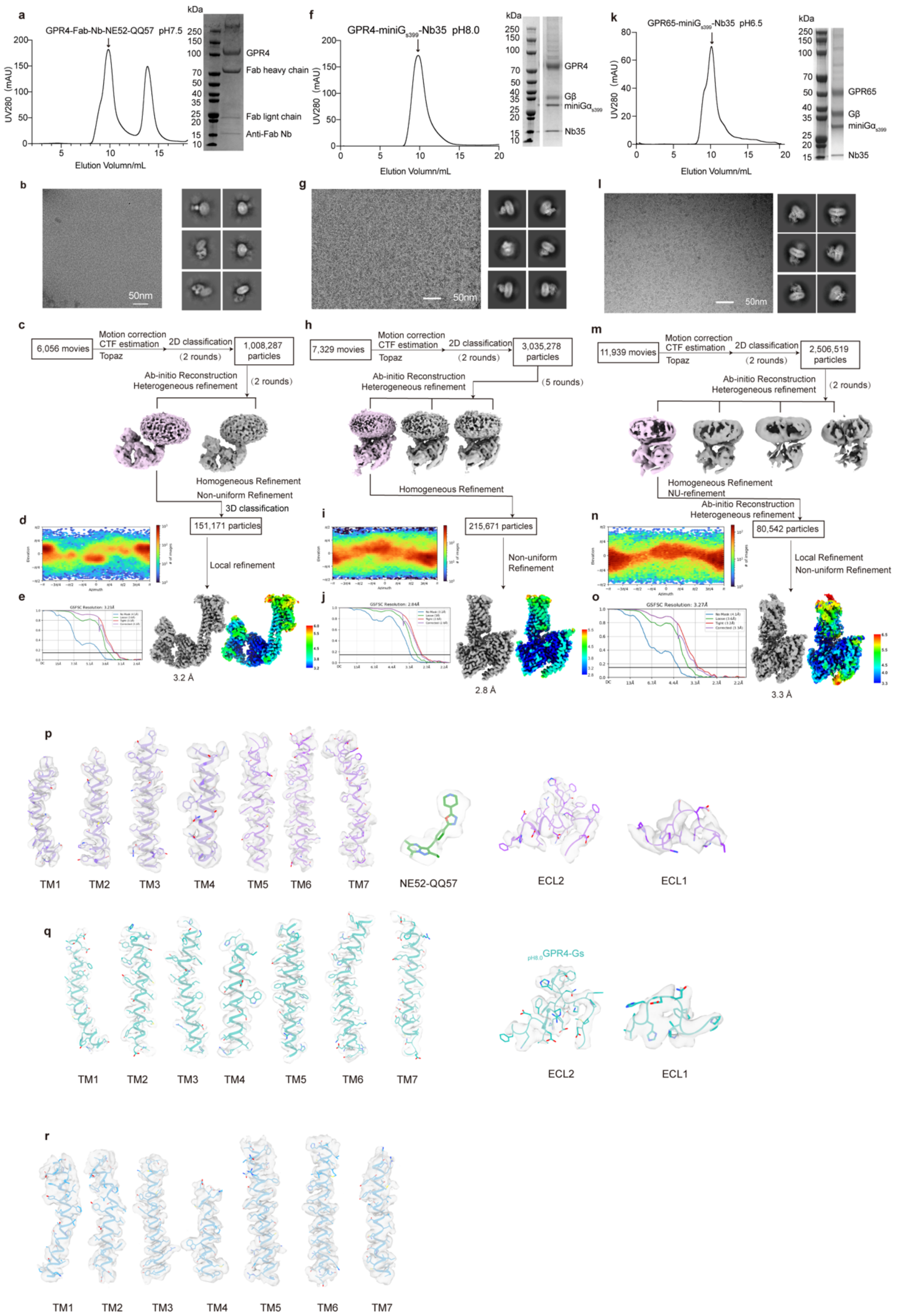

Fig. S5 Sample preparation and cryo-EM data processing of NE52-QQ57-GPR4-Anti-Bril Fab, $_{pH8.0}$GPR4-miniG$_s$-Nb35 and $_{pH6.5}$GPR65-miniG$_s$-Nb35 complexes. **a, f, k,** Superdex200 size-exclusion chromatography elution profiles of the purified

complex samples and SDS-PAGE analysis. **b, g, l,** Representative cryo-EM micrography and selected 2D classification. **c, h, m,** Schematic representation of cryo-EM data processing workflow. **d-e, i-j, n-o,** Angular distribution of the particles used for final reconstruction, cryo-EM maps are colored by local resolution (Å). The Fourier shell correlation (FSC) curves of NE52-QQ57-GPR4-Anti-Bril Fab, **$_{pH8.0}$GPR4-miniG$_s$-Nb35** and $_{pH6.5}$GPR65-miniG$_s$-Nb35. The global resolution of the final processed density map estimated at the FSC = 0.143. **p, q,** Cryo-EM maps and models of TMs and ECLs for NE52-QQ57-GPR4-Anti-Bril Fab (**p**) and $_{pH8.0}$GPR4-miniG$_s$-Nb35 (**q**), complexes, respectively. The density for NE52-QQ57 is shown. **r,** Cryo-EM maps and models of TMs for $_{pH6.5}$GPR65-miniG$_s$-Nb35.
