# Supplementary material for: Structural basis of stepwise proton sensing-mediated GPCR activation
Source: Cell Res. 2025 Apr 11;35(6):423–36. doi: 10.1038/s41422-025-01092-w (PMC12134361; doi:10.1038/s41422-025-01092-w)
Supplement: Supplementary file 6 — Supplementary information, Figure S6 [file 41422_2025_1092_MOESM6_ESM.pdf]

## Supplementary information, Figure S6

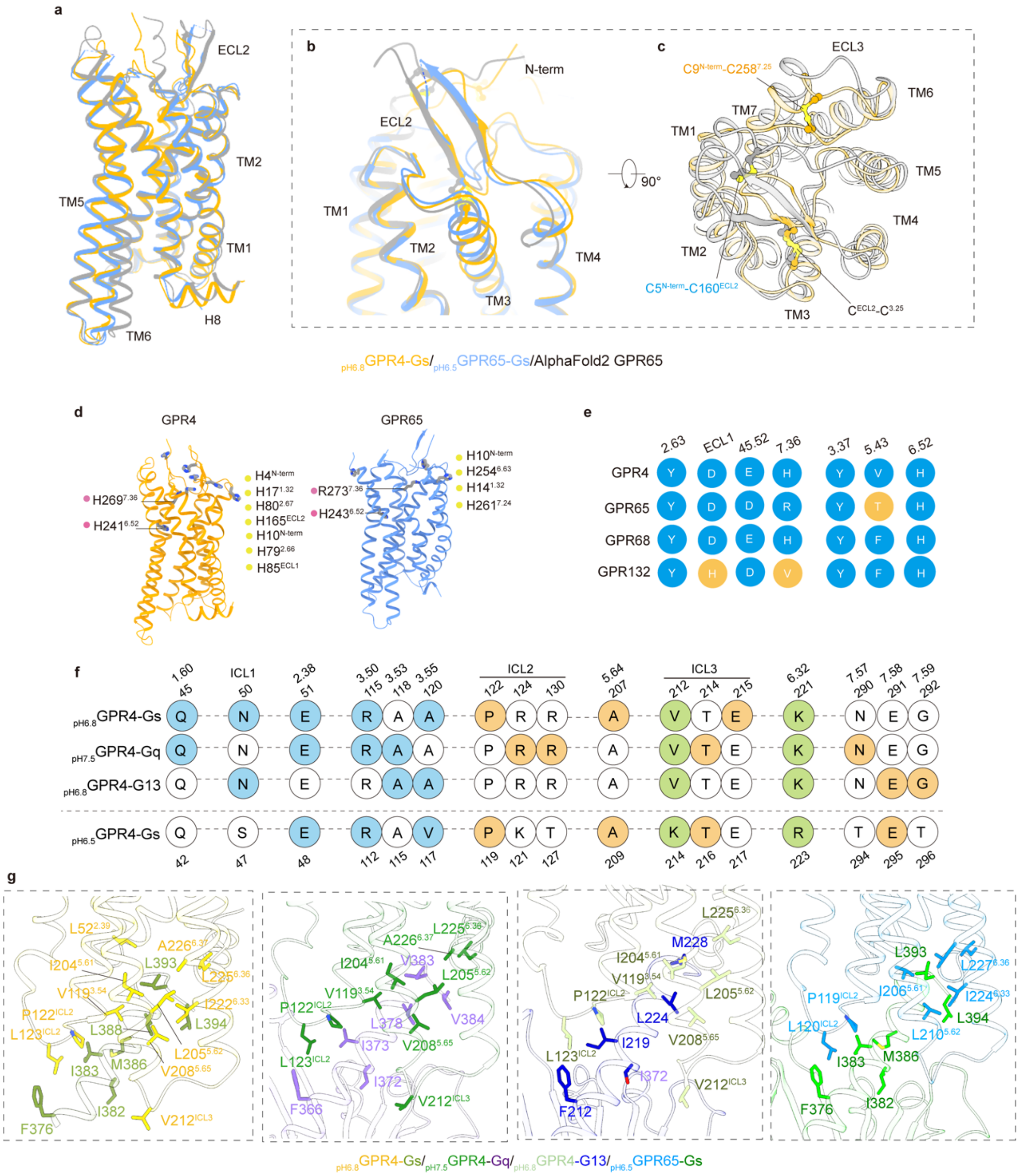


**Fig. S6 Structural comparisons of GPR4 and GPR65 in proton sensing and G protein binding interface. a,** Structural comparison of receptor in $_{pH6.8}$GPR4-Gs with $_{pH6.0}$GPR65-Gs complexes in side view and extracellular view. **b-c,** Structural comparison of ECL2 conformation in GPR4 and GPR65. **d,** Models of GPR4 and GPR65 showing the location of the extracellular His residues in GPR4 and GPR65. **e,** Comparison of the sequence alignment at key residues in proton sensing and activation in GPR4 and GPR65. **f,** Comparison of the sequence alignment at residues involved in

the G protein binding in GPR4 and GPR65. **g,** The detailed interactions between α5 helix and intracellular parts of receptor in GPR4-$G_s$, GPR4-$G_q$, GPR4-$G_{13}$ and GPR65-$G_s$.
