# Supplementary material for: Structural basis of stepwise proton sensing-mediated GPCR activation
Source: Cell Res. 2025 Apr 11;35(6):423–36. doi: 10.1038/s41422-025-01092-w (PMC12134361; doi:10.1038/s41422-025-01092-w)
Supplement: Supplementary file 7 — Supplementary information, Figure S7 [file 41422_2025_1092_MOESM7_ESM.pdf]

Supplementary information, Figure S7

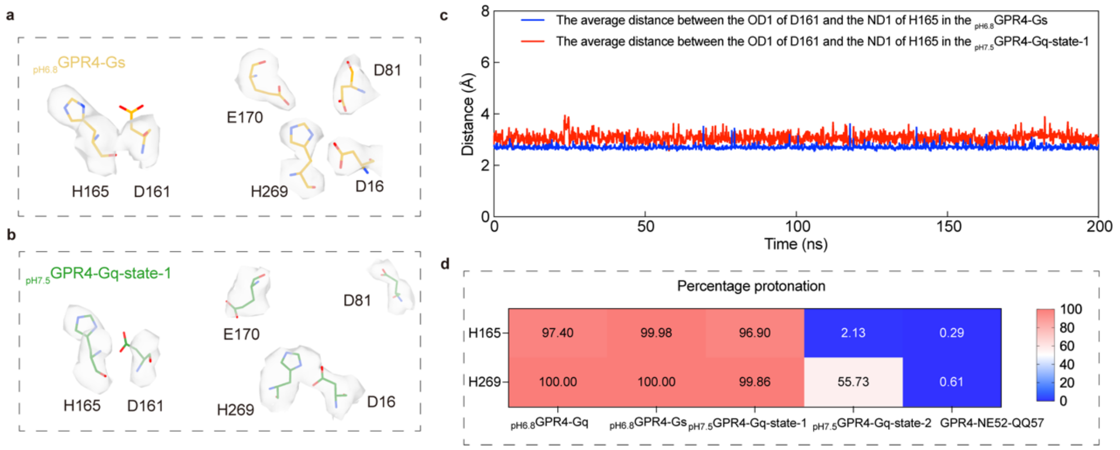

Fig. S7 Cryo-EM density and protonation ratios of residues in GPR4 contributed to proton sensing. a-b, Cryo-EM density of H165^{ECL2} and H269$^{7.36}$ and surrounding residues in the structures of $_{pH6.8}$GPR4-G$_s$ (a) and $_{pH7.5}$GPR4-G$_q$-state-1 (b). **c,** The average distances between OD1 of D161 and ND1 of H165 in $_{pH6.8}$GPR4-G$_s$ (blue) and $_{pH7.5}$GPR4-G$_q$-state-1 (red) structures during triplicate 200 ns MD simulations. **d,** Protonation ratios of H165 and H269 in different GPR4 structures.
