# Supplementary material for: Structural basis of stepwise proton sensing-mediated GPCR activation
Source: Cell Res. 2025 Apr 11;35(6):423–36. doi: 10.1038/s41422-025-01092-w (PMC12134361; doi:10.1038/s41422-025-01092-w)
Supplement: Supplementary file 8 — Supplementary information, Table S1 [file 41422_2025_1092_MOESM8_ESM.pdf]

Supplementary information, Table S1. Cryo-EM data collection, model refinement, and validation statistics.

	$_{pH7.5}$GPR4-DNG$_s$	$_{pH6.8}$GPR4-DNG$_s$	$_{pH6.0}$GPR4-DNG$_s$	$_{pH8.0}$GPR4-miniG$_s$	$_{pH6.8}$GPR4-miniG$_{13}$	$_{pH7.5}$GPR4-miniGs/q state-1	$_{pH7.5}$GPR4-miniGs/q state-2	$_{pH6.8}$GPR4-miniGs/q	GPR4-Anti-Bril Fab-Nb-NE52-QQ57	$_{pH6.5}$GPR65-miniG$_s$
**Data collection and processing**										
Magnification	105,000	105,000	105,000	105,000	130,000	130,000	130,000	130,000	130,000	105,000
Voltage (kv)	300	300	300	300	300	300	300	300	300	300
Electron exposure (e-/Å^2)	60	60	60	60	60	60	60	60	60	60
Defocus range (μm)	-1.2~-2.0	-1.2~-2.0	-1.2~-2.0	-1.2~-2.0	-1.2~-2.0	-1.2~-2.0	-1.2~-2.0	-0.8~-1.5	-0.8~-1.5	-1.2~-2.0
Pixel size (Å)	0.832	0.832	0.832	0.832	0.96	0.96	0.96	0.96	0.96	0.832
Symmetry imposed	C1	C1	C1	C1	C1	C1	C1	C1	C1	C1
Final particles	374,926	314,168	157,993	215,671	171,879	142,072	137,792	276,423	151,171	80542
Map resolution	2.9	3.1	3.1	2.8	3.4	2.9	2.9	2.7	3.2	3.3
FSC threshold	0.143	0.143	0.143	0.143	0.143	0.143	0.143	0.143	0.143	0.143
**Refinement**										
Initial model used (PDB code)	7VUJ	7VUJ	7VUJ	7VUJ	7VUG	7VUJ	7VUJ	7VUJ	8JHC	7VUJ
Map sharping B factor (Å)	88.4	103.4	96.0	66.2	112.7	96.0	97.4	69.8	102.0	99.6
Model-map CC	0.75	0.74	0.71	0.81	0.74	0.77	0.75	0.67	0.73	0.80
Non-hydrogen atoms	8053	7990	7963	8061	8683	8154	8198	7035	7063	7996
Protein residues	1038	1037	1039	1049	1129	1053	1049	920	933	1037
Ligands	0	0	0	0	0	0	0	0	1	0
B-factors										
Protein	66.03	61.71	66.66	59.85	89.20	74.83	77.64	47.92	63.50	89.75
Ligand									67.88	
R.M.S. deviations										
Bond lengths (Å)	0.004	0.003	0.004	0.004	0.003	0.005	0.003	0.004	0.004	0.004
Bond angles (˚)	0.639	0.619	0.661	0.605	0.681	0.740	0.673	0.736	0.677	0.740
Validation										
MolProbity score	2.04	1.88	1.99	1.79	1.82	1.67	1.78	2.12	1.93	1.89
Clash score	14.18	10.42	14.54	13.12	10.62	6.07	8.85	16.52	8.85	15.19
Poor rotamers (%)	0.71	0.48	0.36	0.24	0.00	0.82	0.57	0.83	0.00	0.12
Ramachandran plot										
Favored (%)	94.52	95.20	95.41	97.11	95.98	95.20	95.56	94.18	92.96	96.77
Allowed (%)	5.48	4.80	4.59	2.89	4.04	4.61	4.44	5.38	7.04	3.04
Disallowed (%)	0.00	0.00	0.00	0.00	0.00	0.19	0.00	0.44	0.00	0.20
EMD code	39928	61442	39927	63068	61489	61443	61445	61441	61440	61439
PDB code	8ZCF	9JFW	8ZCE	9LGM	9JHP	9JFX	9JFZ	9JFV	9JFU	9JFT
