# Supplementary material for: Structural basis of stepwise proton sensing-mediated GPCR activation
Source: Cell Res. 2025 Apr 11;35(6):423–36. doi: 10.1038/s41422-025-01092-w (PMC12134361; doi:10.1038/s41422-025-01092-w)
Supplement: Supplementary file 9 — Supplementary information, Table S2 [file 41422_2025_1092_MOESM9_ESM.pdf]

Supplementary information, Table S2. Activity of pH on wild-type GPR4 or GPR65 and mutants, and inhibition of NE52-QQ57 measured by Glo-Sensor assay.

Mutants	pH_{50}±s.e.m[#]	P value	E_{max} (% of WT)	P value	N	Expression (% WT) [$]
GPR4-WT	7.76±0.06		100		3	100
Fusion-GPR4	7.78±0.10	NS	88.28±11.84	NS	3	113.3±4.6
$C9^{N\text{-term}}A$	7.51±0.05	*	74.47±0.59	NS	3	90.0±12.2
$C258^{7.25}A$	7.42±0.09	**	91.23±6.18	NS	3	113.9±4.6
$C90^{3.25}A$	7.83±0.02	NS	47.52±4.87	**	3	75.3±6.8
$C168^{ECL2}A$	7.60 ±0.06	NS	36.27±7.50	***	3	62.7±3.8
$H165^{ECL2}F$	7.46 ±0.02	**	126.87±16.59	NS	3	71.7±3.8
$H269^{7.36}F$	7.53 ±0.02	*	114.40±5.67	NS	3	71.4±7.5
$H241^{6.52}F$	7.31 ±0.04	*	90.20±18.07	NS	3	93.7±5.9
$H165^{ECL2}A/H269^{7.36}A$	6.13 ±0.09	***	29.53±5.43	***	3	35.2±1.9
$H165^{ECL2}F/H269^{7.36}F$	6.85 ±0.01	***	80.11±2.89	NS	3	80.7±5.1
$H165^{ECL2}F/H269^{7.36}F/H241^{6.52}F$	NA	NA	NA	NA	3	54.9±12.9
$D81^{ECL1}N$	8.07 ±0.01	**	111.20±0.90	NS	3	109.9±9.9
$D81^{ECL1}A$	8.14 ±0.03	***	108.46±3.60	NS	3	97.7±15.7
$D161^{ECL2}N$	7.80 ±0.01	NS	123.36±4.32	*	3	113.8±6.9
$D161^{ECL2}A$	7.58 ±0.02	***	165.63±11.06	***	3	105.1±9.2
$D282^{7.49}N$	8.15±0.02	***	76.13±13.70	NS	3	103.2±7.8
$D63^{2.50}N$	7.71 ±0.15	NS	71.88±2.52	*	3	93.5±12.1
L272A	7.20 ±0.14	***	98.42±2.79	NS	3	109.2±20.3
$R115^{3.50}A$	NA	NA	NA	NA	3	93.2±13.8
$P193^{5.50}A$	NA	NA	NA	NA	3	47.7±2.6
$I105^{3.40}A$	7.65 ±0.10	NS	56.84±2.34	*	3	119.7±6.5
Y98A	7.52 ±0.12	*	90.39±13.11	NS	3	96.7±13.1
$F97^{3.32}A$	7.32 ±0.04	***	94.57±12.27	NS	3	121.4±12.6

Mutants	pH_{50}±s.e.m[#]	P value	E_{max} (% of WT)	P value	N	Expression (% WT) [$]
GPR65-WT	6.90±0.04		100		3	100
$C5^{N\text{-term}}A$	6.47±0.03	**	44.44±4.38	***	3	80.7 ±3.0
$C160^{ECL2}A$	6.19 ±0.04	***	33.62±4.92	***	3	74.0 ±2.8
$C87^{3.25}A$	6.48±0.12	**	6.49 ±1.62	***	3	50.5 ±6.4
$C170^{ECL2}A$	6.70±0.02	NS	20.69 ±3.38	***	3	68.7 ±2.8
$D172^{ECL2}A$	NA	NA	NA	NA	3	94.6 ±8.5
$R273^{7.36}A$	NA	NA	NA	NA	3	81.6±5.2
$H243^{6.52}F$	NA	NA	NA	NA	3	53.7±4.4
$R112^{3.50}A$	NA	NA	NA	NA	3	54.9±2.9
$D60^{2.50}A$	6.67±0.09	NS	14.23±3.69	***	3	95.1±0.1
$D286^{7.49}A$	6.78 ±0.09	NS	9.28±0.93	NS	3	89.5±0.1
$D78^{ECL1}A$	6.56 ±0.05	**	62.35±10.21	**	3	154.9±12.6
$H10^{N\text{-term}}A$	6.46 ±0.05	**	40.63±7.36	***	3	113.3±1.0
$F98^{3.36}A$	6.81±0.08	NS	51.50±11.79	***	3	38.5±2.7
$F239^{6.48}A$	7.02±0.06	NS	45.01±10.06	**	3	108.6±15.1
F235A	6.95±0.06	NS	59.32±14.50	*	3	81.2±5.3
Y272A	6.34±0.16	**	36.63±7.31	**	3	113.8±6.9
F242A	6.70±0.15	**	47.41±5.90	**	3	95.3±7.7

Mutants	pIC_{50}±s.e.m[#]	P value	N	Expression (% WT) [$]
GPR4-WT	7.20±0.08		7	100
$F167^{7.36}A$	6.74±0.03	*	3	93±5.2
$L272^{7.39}A$	6.43±0.19	***	3	89±7.6
$Y76^{2.63}A$	6.07±0.07	***	3	102±5.5
$D81^{ECL1}A$	6.96±0.07	ns	3	97.7±15.7
$Y24^{1.39}F$	5.42±0.19	***	3	106.9±14.8
$Y24^{1.39}A$	5.38±0.13	***	3	75.7±3.6
$L70^{2.57}A$	6.17±0.06	***	3	97.9±15.9
$W73^{2.60}F$	5.92±0.04	***	3	121.1±10.7
$W73^{2.60}A$	5.35±0.24	***	3	107.3±6.5
$F77^{2.64}A$	6.16±0.11	***	3	54.9±7.8
$H269^{7.36}F$	8.94±0.49	***	3	71.4±7.5

[#]Data are mean ± s.e.m. from at least three independent experiments. *p<0.05, **p<0.01, ***p<0.001 by one-way analysis of variance followed by Dunnett's post-test compared to the response of wild type. NS, not significant; NA (not activated) indicates that the value cannot be fitted by non-linear regression in GraphPad Prism. Data are mean ± s.e.m. from three independent experiments.

[$]Protein expression levels of GPR4 or GPR65 constructs at the cell surface were determined in parallel by flow cytometry with an anti-Flag antibody (Sigma) and reported as per cent compared to the wild type from three independent measurements performed in technical duplicate.
