# Supplementary material for: Structural basis of stepwise proton sensing-mediated GPCR activation
Source: Cell Res. 2025 Apr 11;35(6):423–36. doi: 10.1038/s41422-025-01092-w (PMC12134361; doi:10.1038/s41422-025-01092-w)
Supplement: Supplementary file 14 — Supplementary information, Video legend [file 41422_2025_1092_MOESM14_ESM.pdf]

Supplementary information, Video **S1**

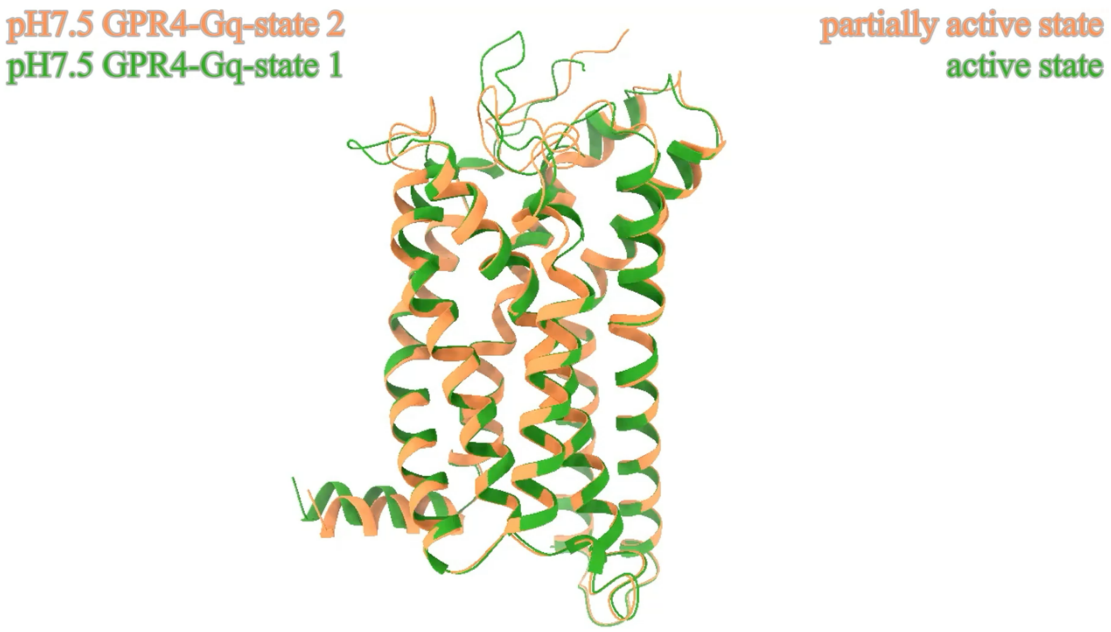

Video **S1.** The conformational changes of the extracellular loops between $_{pH7.5}$GPR4-Gq-state-1 and $_{pH7.5}$GPR4-Gq-state-2 structures.

Supplementary information, Video **S2**

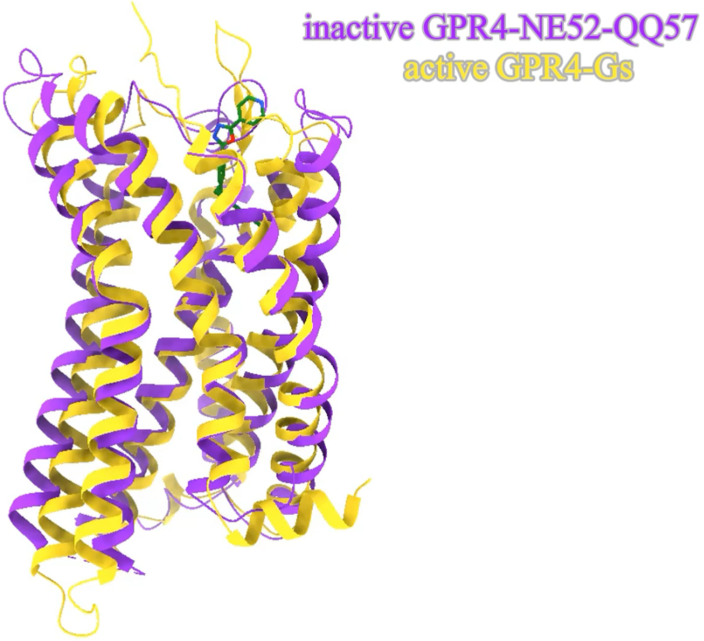

Video **S2.** The conformational changes of the extracellular loops between $_{pH6.8}$GPR4-Gs and inactive NE52-QQ57-GPR4 structures.

Supplementary information, Video **S3**

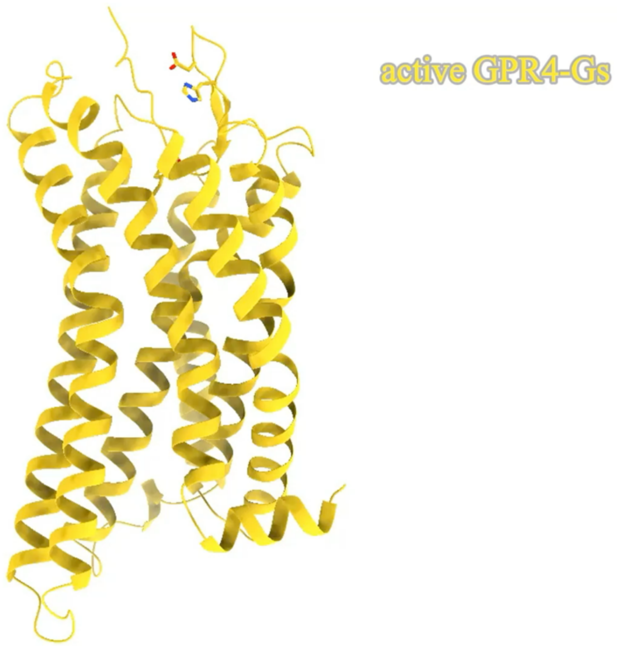

Video **S3.** The conformational changes of the key proton sensing residues between $_{pH6.8}$GPR4-Gs and inactive NE52-QQ57-GPR4 structures.

Supplementary information, Video **S4**

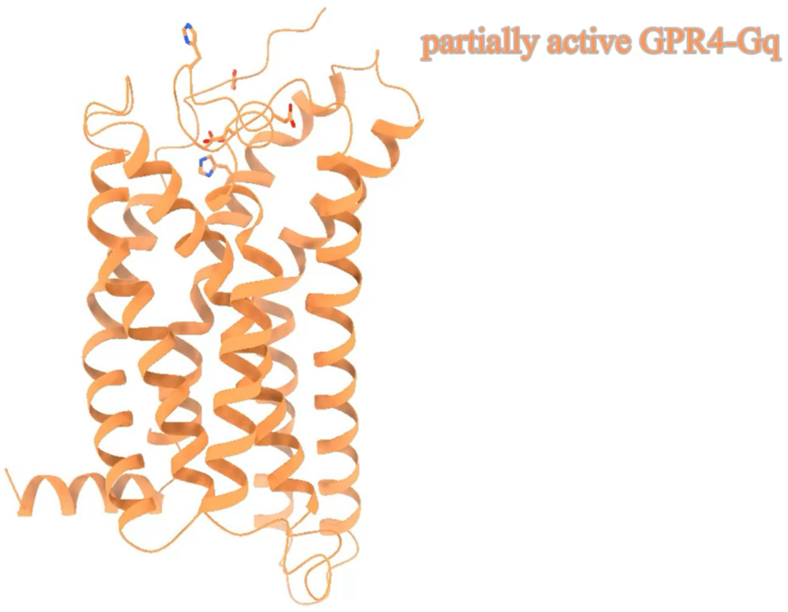

Video **S4.** The conformational changes of the key proton sensing residues between $_{pH7.5}$GPR4-Gq-state-1 and $_{pH7.5}$GPR4-Gq-state-2 structures.
